# Artificial Intelligence Integrated Smart Medical Imaging Lab Framework for Enhanced Diagnosis and Treatment of Pandemic‐Prone Diseases

**DOI:** 10.1002/hsr2.71972

**Published:** 2026-03-08

**Authors:** Aditika Tungal, Prabhsimran Singh, Kuldeep Singh, Pankaj Deep Kaur, Salil Bharany, Ruby Pant, Ajay Kumar, Ateeq Ur Rehman, Seada Hussen

**Affiliations:** ^1^ Department of Computer Engineering & Technology Guru Nanak Dev University Amritsar India; ^2^ Department of Electronics Technology Guru Nanak Dev University Amritsar India; ^3^ Department of Engineering and Technology Guru Nanak Dev University, Regional Campus Jalandhar; ^4^ Chitkara University Institute of Engineering and Technology Chitkara University Rajpura India; ^5^ Department of Mechanical Engineering Uttaranchal University Dehradun India; ^6^ Department of Mechanical Engineering Noida Institute of Engineering and Technology (NIET) Greater Noida India; ^7^ School of Computing Gachon University Seongnam‐si Republic of Korea; ^8^ Department of Electrical Power Adama Science and Technology University Adama Ethiopia

**Keywords:** blood test, CNN, COVID‐19, CT‐scan, explainable AI, healthcare, machine learning, severity, smart laboratory diagnosis, X‐ray

## Abstract

**Background:**

The COVID‐19 pandemic has caused massive devastation worldwide, and its effects still persist. Managing the early stages was difficult, but scientists worked tirelessly to control it. The emergence of variants continues to pose a threat, raising doubts about the capability of the healthcare system. Healthcare practitioners have faced immense strain under a massive patient load, while delays in testing have caused deaths due to untimely treatment. Moreover, relying only on RT‐PCR testing is insufficient because of its diagnostic errors.

**Materials and Methods:**

To address these challenges, this study introduces a Smart Imaging Lab Framework for hospitals. The approach uses a convolutional neural network (CNN) model to carry out rapid X‐ray and CT‐scan assessments of emergency patients showing severe symptoms, following RT‐PCR testing. In addition, blood tests help determine the severity of infection. Patients in critical condition are transferred to intensive care units, while those with milder cases remain in general wards.

**Results:**

The framework uses a 16‐layer CNN framework for X‐ray and CT‐scan imaging, achieving 99.02% and 98.49% accuracy, respectively. Severity assessment with Extra Randomized Trees reached 98.00% accuracy.

**Discussion:**

These findings highlight the potential of the system to be adopted in hospitals, enabling regular health monitoring and timely intervention. In addition, explainable AI XAI tools like Grad‐CAM increase transparency by highlighting the lung regions most relevant to the diagnosis.

**Conclusion:**

The study demonstrates the potential of artificial intelligence, internet of things, and cloud computing to address future pandemic‐prone diseases.

## 1 Introduction

The COVID‐19 pandemic has undoubtedly left a lasting impact on people worldwide. Despite more than 5 years since its onset, new variants such as Omicron, Delta, Alpha, Beta, and Gamma continue to emerge, some of which are more contagious and transmissible than others [1]. Concurrently, individuals have been grappling with numerous health issues. As reported by the World Health Organization (WHO), various clinical symptoms have been linked to SARS‐CoV‐2 [2, 3]. Given their overlap with common diseases, clinically validated laboratory tests are essential for diagnosis.

The predominant method for detecting the 2019‐nCoV virus is genome testing through RT‐PCR [4]. However, this technique is time‐consuming, sometimes prone to false negatives, and many countries have faced shortages of testing kits [5]. In response, alternative diagnostic methods have been explored. Medical imaging methods such as chest X‐rays and CT‐scans, play a crucial role in detecting COVID‐19 and related pulmonary diseases [6]. The devastating impact of the pandemic led scientists to explore the correlation between blood parameters and disease severity [7]. The Complete Blood Count (CBC), a widely used and cost‐effective test, provides important biomarkers such as red and white blood cells, hemoglobin, platelet count, C‐reactive protein, d‐dimer, lymphocytes, and neutrophils [8]. These markers help differentiate between Severe and Non‐severe cases.

Relying solely on RT‐PCR is insufficient; hence, advanced approaches are needed to reduce the burden on laboratories and healthcare workers. Artificial intelligence (AI), especially machine learning (ML) and deep learning (DL), can help speed up the identification process [9, 10, 11, 12, 13]. Building on this, automation of smart laboratories is essential to manage the influx of patients and minimize contamination. Integrating services such as X‐rays, CT‐scans, and blood tests on one platform using AI‐based solutions reduces manual workload and provides a comprehensive examination of suspected cases. However, most existing efforts have focused on these data sources separately, limiting practical applicability [14, 15, 16, 17]. A unified framework combining imaging and blood‐based diagnostics can therefore deliver faster, more reliable, and scalable outcomes during pandemics (refer to Section S1 of the supplementary file for related work).

In this regard, the present study establishes automated laboratories that integrate diagnostic procedures. Along with RT‐PCR validation, patients undergo X‐rays for visualization of lung infections, followed by routine blood tests to gauge severity. ML and DL classifiers operate in the background to enhance laboratory functionality. This hybrid approach, combining virus identification and severity assessment, ensures all tests are conducted in one setting with reports promptly delivered to healthcare providers. With the emergence of new variants, this intelligent approach minimizes the need for patients to visit separate labs. Beyond COVID‐19, it can also be applied to other diseases, ensuring timely identification and accurate diagnosis. The major contributions of the present work include:

- Automation of laboratories for diagnosing COVID‐19, enhancing detection accuracy in a shorter timeframe and minimizing manual workload for medical staff.
- Classification of patients as COVID‐19 or Non‐COVID‐19 using X‐ray and CT‐scan, and as Severe or Non‐Severe based on blood reports.
- High classification performance with accuracies of 99.02% for X‐ray classification, 98.49% for CT‐scan classification, and 98.00% for severity determination using blood markers.
- A unified framework integrating imaging and blood‐based diagnostics into one smart lab system, achieving high accuracy and reducing multiple lab visits.

The novelty of this work lies in its unified framework, where a single CNN architecture is employed for both X‐ray and CT‐scan images, and ML models are used to assess severity from blood parameters. Unlike earlier studies that considered imaging or blood markers separately, this approach integrates both into one smart lab system, achieving high accuracy and reducing the need for multiple lab visits. In addition, XAI based Grad‐CAM visualization is applied to highlight critical lung regions influencing predictions, further enhancing interpretability and clinical relevance.

This paper is organized into several sections. Section II outlines the proposed hybrid smart lab setup. Section III presents the results obtained. Section IV compares the present study with previous research and discusses limitations and future applications. Finally, Section V concludes the paper.

## 2 Materials and Methods

**Figure 1:**
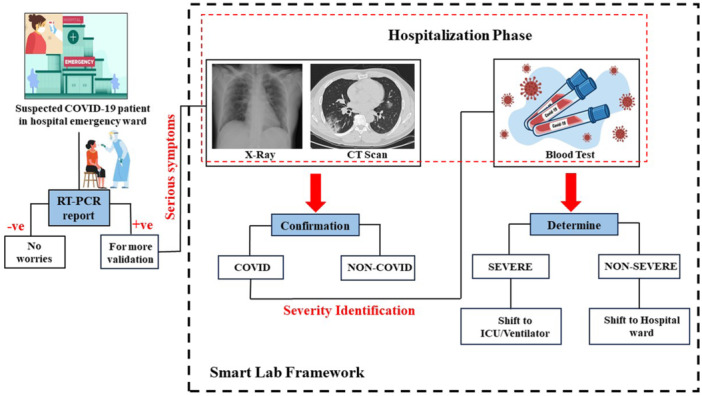
Overview of the proposed Smart Lab Framework.

This section outlines the methodology of the proposed framework shown in Figure 1. When a suspected patient reaches the emergency ward, vitals are checked and an RT‐PCR test is performed which is the gold standard for COVID‐19 detection [18]. However, due to misclassification rates, RT‐PCR alone is insufficient [19]; therefore, complementary techniques are incorporated. If a patient tests positive and exhibits serious symptoms, chest radiographs and CT‐scans are conducted to assess infection and support confirmation. Once infection is confirmed, severity is determined using blood test values, where deviations from normal ranges indicate disease intensity [20].

Patients with critical symptoms are admitted to the Intensive Care Unit (ICU), while less severe cases are treated in hospital wards. The smart lab framework integrates RT‐PCR, imaging, and blood tests with AI‐based ML and DL methods to ensure accurate diagnosis and severity assessment. Timely execution of these steps enables early detection and effective treatment, providing an efficient approach to managing pandemic‐prone diseases.

**Figure 2:**
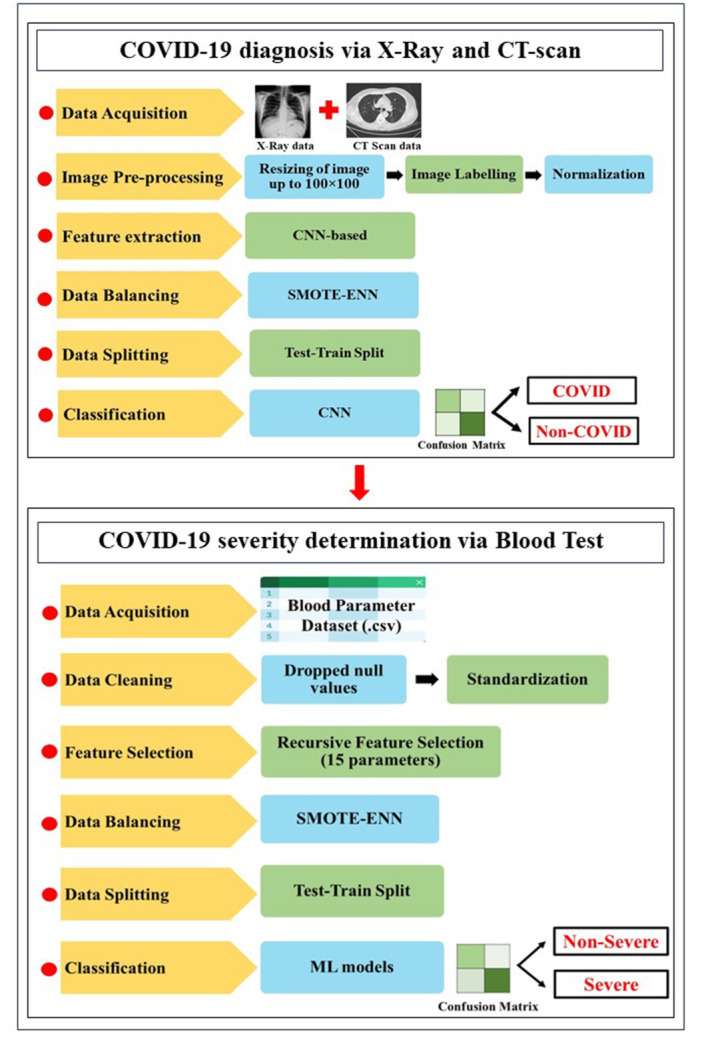
Illustration of the proposed methodology.

The development of the smart lab environment involves several steps. COVID‐19 identification begins with analysis of X‐ray and CT‐scan data, including image acquisition, pre‐processing, and data balancing. Classification is then performed using CNN and hybrid ML models. After cases are identified, severity is assessed through blood tests. A publicly available blood dataset is cleaned, relevant clinical biomarkers are selected, and balancing procedures are applied. Multiple ML models are then trained and tested to predict severity levels. Figure 2 provides a sequential overview of the methodology, with details described in the following sub‐sections.

### 2.1 Data Description

**Figure 3:**
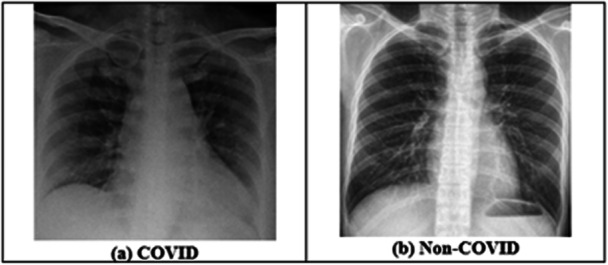
X‐ray images given in the dataset.

**Figure 4:**
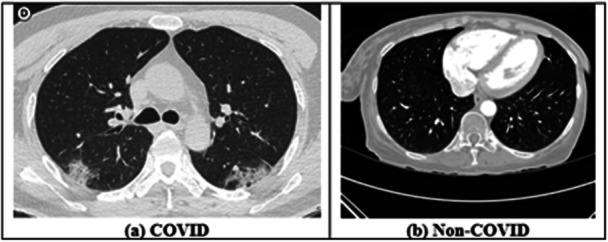
CT‐scan images from the dataset.

The study employs the ‘Extensive COVID‐19 X‐ray and CT Chest Images Dataset’ provided by Menoufia University [21, 22] to analyze X‐ray and CT‐scan images for the detection of COVID‐19. The dataset contains 9544 X‐ray and 8055 CT‐scan images of COVID and Non‐COVID cases. These images are illustrated in Figures 3 and 4. In addition, the ‘BIGDATA‐COVID19’ blood marker dataset [21] was used for severity assessment. It contains 4995 records with 22 features, where severity serves as the output variable. The data were collected from 1218 patients admitted to the Emergency Department of San Raffaele Hospital (OSR), Milan, Italy, between February 19 and May 31, 2020 (see Section S2 of the supplementary file for more details).

### 2.2 Pre‐Processing

For imaging data, images were resized to 100×100 pixels and normalized by dividing them by 255 [23] to improve the computational complexity of the proposed approach. Similarly, the prognostic blood dataset was processed by removing Null entries to avoid bias and standardized to ensure equal scaling.

### 2.3 Feature Extraction and Feature Selection

**Figure 5:**
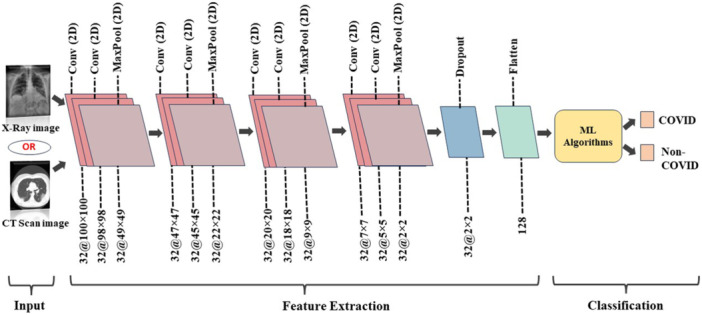
CNN as a feature extractor.

**Figure 6:**
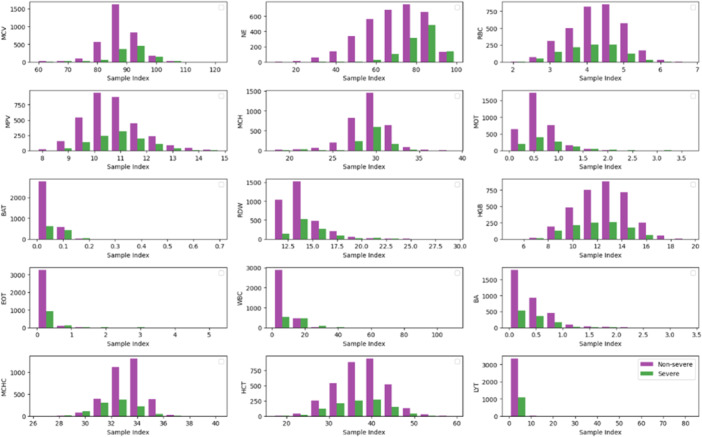
Relevant features obtained after applying RFE.

In this study, CNN was employed to extract features from X‐ray and CT‐scan images, whereas RFE was applied to the blood dataset. A pre‐trained 16‐layer CNN with 8 convolutional layers, 4 max‐pooling layers, 1 dropout, and 1 flatten layer (Figure 5) was employed, and the extracted features were passed to ML algorithms for COVID and Non‐COVID classification. For the blood dataset, RFE ranked features by importance and selected 15 relevant parameters, mentioned in Table 1. These features are shown in Figure 6. The histograms represent severity distribution, where 0 refers to Non‐severe cases and 1 refers to severe cases. Each subplot represents one feature, with classes distinguished by color, the x‐axis shows sample indices and the y‐axis represents feature values.

**Table 1:** List of blood parameters selected by the RFE.

Blood parameter	Abbreviation
Mean corpuscular volume	MCV
Neutrophil A	NE
Red blood cells	RBC
Mean platelet volume	MPV
Mean corpuscular hemoglobin	MCH
Monocyte B	MOT
Basophil B	BAT
Erythrocyte distribution width	RDW
Hemoglobin	HGB
Eosinophil B	EOT
White blood cell	WBC
Basophil A	BA
Mean corpuscular hemoglobin concentration	MCHC
Hematocrit	HCT
Lymphocyte B	LYT
Output parameter	Severity

### 2.4 Data Balancing Using SMOTE‐ENN

In the X‐ray dataset, the COVID class had 4,044 images and the Non‐COVID class had 5500 images. After applying SMOTE‐ENN, counts became 4960 and 4186, respectively. In the CT‐scan dataset, COVID images were 5427 and Non‐COVID were 2628. After balancing they became 5300 and 4659 respectively. SMOTE‐ENN improved generalization by oversampling the minority class [24] and removing noisy samples, while models trained on unbalanced data performed worse. In the blood dataset, there were 3342 Non‐severe cases and 1088 severe cases. After balancing with SMOTE‐ENN, Non‐severe decreased to 2378 and severe increased to 3107.

### 2.5 Proposed Architecture for the Determination of COVID‐19 Among Patients

**Figure 7:**
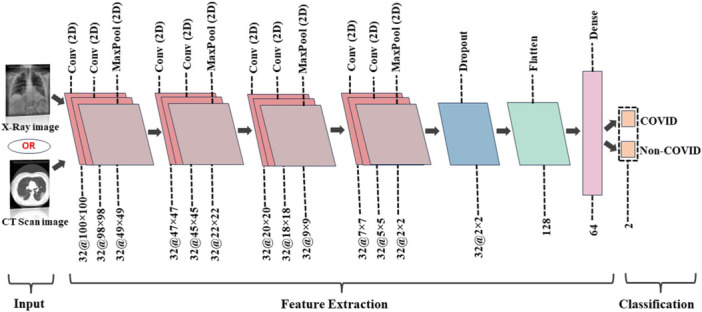
Proposed CNN architecture for the determination of COVID‐19 patients.

After preprocessing and class balancing, model training was performed using ML and DL techniques. For image classification, a unified 16‐layer CNN was applied to both X‐ray and CT‐scan images, providing a single efficient model. The architecture, shown in Figure 7, consists of 8 Conv2D layers, 4 MaxPooling2D layers, 1 Dropout layer, 1 Flatten layer, 1 Dense layer, and 1 output layer for binary classification. The input shape is (100, 100, 3), with each Conv2D layer using 32 filters and 3 × 3 kernels.

Several characteristics of the proposed CNN are summarized in Table 2. As shown in Figure 7, the model has two output classes, COVID and Non‐COVID. ReLU activation was used in all convolutional and dense layers. Training experiments tested learning rates of 0.001, 0.01, and 0.1, with 0.0001 giving the best performance. The Adam optimizer was used with a batch size of 16 over 100 training epochs. These settings, detailed in Table 2, produced optimal outcomes. The CNN also functioned effectively as a feature extractor, as described in Figure 5 (refer to Sections S3 and S4 of the supplementary file for further details on the CNN). Experiments were conducted on a system with an NVIDIA GeForce RTX 3060 GPU, AMD Ryzen 7 5800H processor, and 16 GB RAM, which enabled efficient preprocessing, training, and evaluation through parallel GPU computation.

**Table 2:** CNN model parameter details.

Parameter	Value
Number of classes	2
Activation function	ReLU
Activation function for classification layer	Softmax
Pooling	MaxPooling2D
Filter size	32
Kernel size	3×3
Learning rate	0.0001
Optimizer	adam
Batch size	16

Furthermore, Gradient‐weighted Class Activation Mapping (Grad‐CAM), an explainable AI (XAI) technique was used to visually show the areas of the lungs that influenced model predictions. This approach generates heatmaps of COVID‐19‐related lung infection, highlighting regions most relevant for classification [25]. By incorporating Grad‐CAM, the model highlights decision‐related lung regions, aiding clinical understanding and use.

## 3 Results

This section outlines the outcomes of the proposed smart lab framework, which performs two key tasks by detecting COVID‐19 through X‐ray and CT‐scan images and by assessing severity using blood parameters. For imaging data, hybrid ML models were evaluated alongside the CNN, while for the blood dataset, multiple ML models were applied as described earlier. All models were assessed with stratified 10‐fold cross‐validation (CV), using 90/10 train‐test splits per fold, and metrics were reported as fold averages. The following sub‐sections summarize the results for each task.

### 3.1 Determination of COVID‐19 Among the Patients

Accurate identification of COVID‐19 cases requires careful and efficient testing. For patients with severe symptoms, this study employs X‐ray and CT‐scan based approaches to enable visual assessment of infection. Multiple ML models such as histogram‐based gradient boosting (HGB), extremely randomized tree (ERT), gradient boosting (GB), and decision tree (DT) were trained alongside a unified CNN model to classify cases as COVID‐19 and Non‐COVID‐19. Performance was evaluated using accuracy, sensitivity, specificity, precision, false discovery rate (FDR), and prediction time (ms/image).

For X‐ray images, the CNN model was evaluated alongside HGB, ERT, GB, and DT classifiers. Table 3 presents the performance metrics, showing that the CNN reached 99.02% accuracy, with HGB at 95.74%, ERT at 94.86%, GB at 92.90%, and DT at 87.32%. Similarly, CT‐scan images were analyzed since they play a crucial role in assessing patient health during COVID‐19. Table 4 reports the outcomes, indicating that the CNN attained 98.49% accuracy, while HGB, ERT, GB, and DT recorded 95.38%, 95.08%, 92.97%, and 89.56%, respectively (see Sections S5 and S6 of the supplementary file for detailed visualization of X‐ray and CT‐scan results).

**Table 3:** Performance assessment for X‐ray images.

Classifiers	Accuracy (%)	Sensitivity (%)	Specificity (%)	Precision (%)	FDR (%)	Prediction time (ms/image)
CNN	99.02	99.02	99.02	99.00	1.00	2.037
HGB	95.74	95.57	95.57	95.85	4.15	2.115
ERT	94.86	94.71	94.71	94.93	5.07	2.116
GB	92.90	92.56	92.56	93.19	6.81	2.098
DT	87.32	87.19	87.19	87.22	12.78	2.097

**Table 4:** Performance assessment for CT‐scan images.

Classifiers	Accuracy (%)	Sensitivity (%)	Specificity (%)	Precision (%)	FDR (%)	Prediction time (ms/image)
CNN	98.49	98.49	98.49	98.50	1.50	2.173
HGB	95.38	95.31	95.31	95.47	4.53	2.247
ERT	95.08	94.95	94.95	95.35	4.65	2.264
GB	92.97	92.82	92.82	93.29	6.71	2.257
DT	89.56	89.53	89.53	89.55	10.45	2.234

**Figure 8:**
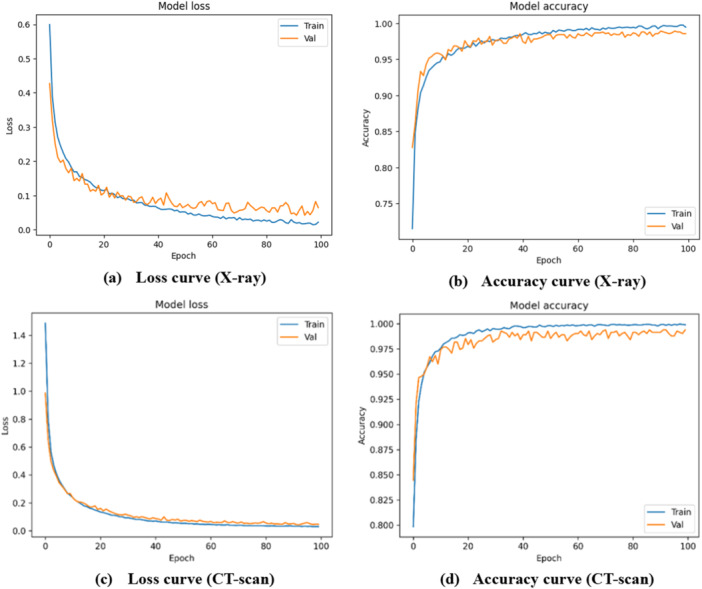
CNN performance (a) Loss curve (X‐ray), (b) Accuracy curve (X‐ray), (c) Loss curve (CT‐scan) and (d) Accuracy curve (CT‐scan).

**Figure 9:**
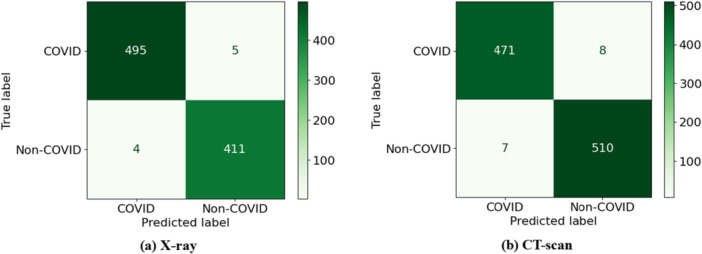
Confusion Matrix of CNN (a) X‐ray (b) CT‐scan.

**Figure 10:**
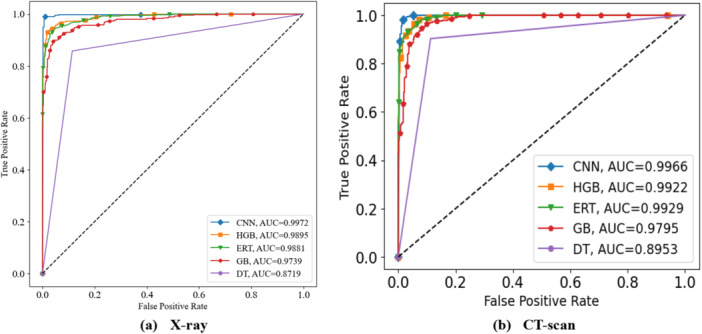
ROC curve (a) X‐ray (b) CT‐scan.

Furthermore, to assess the effectiveness of the proposed model, sensitivity, specificity, precision, and false discovery rate (FDR) were evaluated [26]. Based on these metrics, the CNN achieved the best performance (refer to Sections S5 and S6 of the supplementary file for class‐wise results of X‐ray and CT‐scan data). In addition, the training of the developed CNN model was assessed using loss and accuracy curves, shown in Figure 8a,b for X‐ray data and Figure 8c,d for CT‐scan images, respectively. Moreover, Figure 9a shows the confusion matrix of the CNN model for X‐ray data with two classes, COVID and non‐COVID, while Figure 9b presents the corresponding matrix for CT‐scan data. Model performance across thresholds was further examined using receiver operating characteristic (ROC) curves and area under the curve (AUC). Figure 10a displays the ROC curves for all classifiers including HGB, ERT, GB, DT, and CNN on X‐ray data, whereas Figure 10b presents the ROC curves for the same models on CT‐scan data. The corresponding AUC values show that CNN outperforms the others in classifying patients as COVID or Non‐COVID.

**Figure 11:**
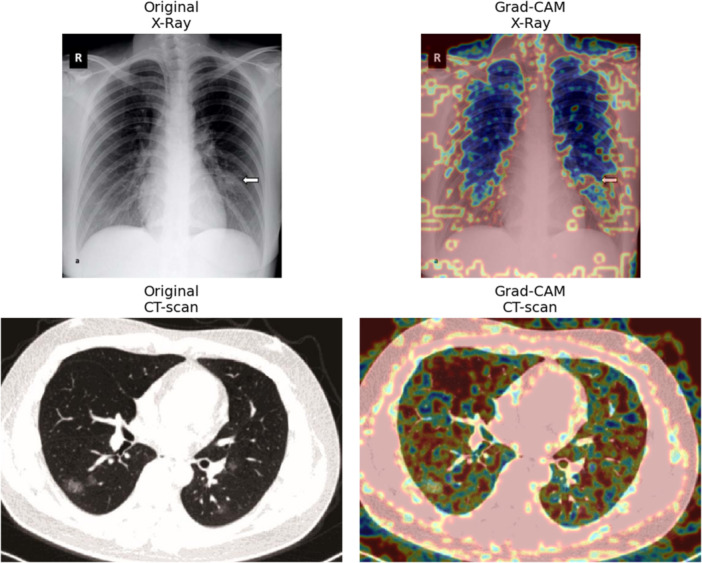
Grad‐CAM visualizations for X‐ray and CT‐scan images.

Furthermore, to enhance the visibility, the Gradient‐weighted Class Activation Mapping (Grad‐CAM) technique, an explainable AI (XAI) method, was applied. The colors in the Grad‐CAM images highlight abnormal lung regions most relevant to model decisions. Figure 11 shows representative overlays, demonstrating that the network focuses on clinically meaningful patterns, improving transparency and supporting clinical adoption.

### 3.2 Determination of Severity Among the Patients Based on Blood Tests

**Figure 12:**
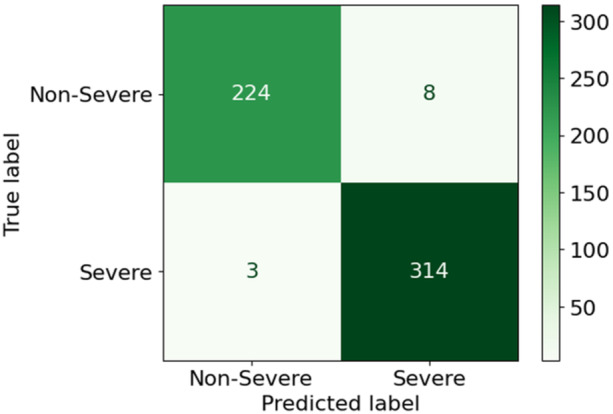
Confusion matrix of the ERT model in case of blood dataset.

**Figure 13:**
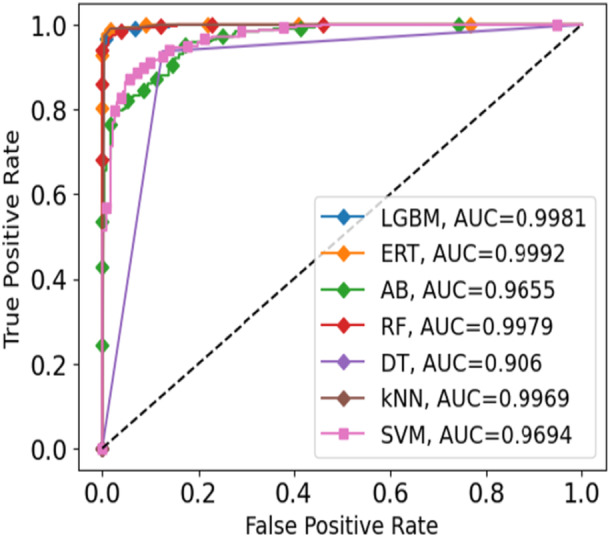
ROC plot for various ML models for the blood dataset.

Following COVID‐19 detection via X‐ray and CT‐scan, disease severity was evaluated using blood markers. Patients were grouped as Severe or Non‐severe with the help of several ML models, including Extremely Randomized Trees (ERT), Light Gradient Boosting Machine (LGBM), Random Forest (RF), K‐Nearest Neighbor (kNN), Decision Tree (DT), Support Vector Machine (SVM), and Adaboost (AB), as shown in Table 5. ERT gave the best accuracy of 98.00%, followed by LGBM at 97.81%, RF at 97.45%, and kNN at 96.36%, while DT, SVM, and AB reached 91.07%, 90.16%, and 87.98%, respectively. Prediction time (ms/sample) for all models is also provided in Table 5 (see Section S7 of supplementary file for class‐wise results). In addition, the confusion matrix was used to compare actual and predicted values. Figure 12 shows the confusion matrix for the ERT model which is divided into TP, FP, FN, and TN. Finally, the severity classification models were compared using ROC and AUC values, evaluated across different thresholds and shown in Figure 13.

**Table 5:** Performance assessment for blood data.

Classifiers	Accuracy (%)	Sensitivity (%)	Specificity (%)	Precision (%)	FDR (%)	Prediction time (ms/sample)
ERT	98.00	97.80	97.80	98.10	1.90	0.024
LGBM	97.81	97.82	97.82	97.71	2.29	0.025
RF	97.45	97.16	97.16	97.64	2.36	0.018
kNN	96.36	95.81	95.81	96.83	3.17	0.103
DT	91.07	90.60	90.60	91.07	8.93	0.002
SVM	90.16	89.81	89.81	90.00	10.00	0.102
AB	87.98	87.74	87.74	87.66	12.34	0.017

Therefore, the proposed framework achieved two key objectives: distinguishing COVID from Non‐COVID cases and classifying patients by severity. Severe cases require prompt intervention, including transfer to critical care or intensive care units for continuous monitoring and timely assessment.

## 4 Discussions

### 4.1 Comparative Model Complexity and Model Deployability

The proposed 16‐layer CNN contains ~74k trainable parameters, far fewer than heavy transfer learning models such as VGG16, DenseNet121, and MobileNet, yet achieves 99.02% accuracy on X‐rays and 98.49% on CT‐scans (Table 6). Its compact size minimizes memory use and speeds up training and inference, enabling real‐time deployment in resource‐constrained hospitals. Moreover, with prediction times of ~2 ms per image (Tables 3, 4), the model demonstrates practical efficiency for clinical applications.

**Table 6:** Comparison of DL models by trainable parameters.

DL model	Trainable parameters
Proposed CNN (16‐layer)	~ 74,018
VGG‐16 [27]	~ 138 million
MobileNet [28]	~ 4.2 million
DenseNet121 [29]	~ 8 million

### 4.2 Comparison with Other Studies

Following the model‐size benchmarking in Table 6, this section compares prominent studies that used the same dataset as the proposed framework. Table 7 compiles details of image counts, preprocessing methods, classifiers, and accuracy achieved. Some studies focused solely on X‐ray images [30], while others used only CT‐scan images [32]. Preprocessing measures varied across these works, including image resizing, labeling, normalization, feature extraction, data balancing, and classification.

**Table 7:** Comparison of the prominent research works for the ‘Extensive COVID‐19 X‐ray and CT Chest Images Dataset’.

Study, Year	No. of images	Methodology	Classifier	Accuracy
Ismail et al., 2021 [30]	X‐ray: 4000 (Covid = 2000, Non‐Covid = 2000)	Image labeling; feature extraction; data splitting; hyperparameter tuning	InceptionV3	X‐ray: 96.00%
El‐Shafai et al., 2022 [31]	X‐ray: 1000 CT‐scan: 1000	—	CNN (SGDM + ReLU optimizer)	X‐ray: 91.67%;
				CT‐scan: 100.00%;
Mohbey et al., 2022 [32]	CT‐scan: 5000	Image resizing to 224×224 RGB	VGG	CT‐scan: 95.00%
Ravi et al., 2022 [22]	X‐ray: 9544 CT‐scan: 8055	Image resizing to n×n pixels; normalization; CNN based feature extraction; feature fusion; kernel PCA; dimensionality of extracted features; t‐SNE for feature visualization; stacking	EfficientNet	X‐ray: 99.00%
				CT‐scan: 99.00%
Hayat et al., 2023 [33]	X‐ray: 9544 CT‐scan: 8055	Image resizing to 224×224 pixels; min‐max normalization	SCovNet	X‐ray: 97.62%
				CT‐scan: 97.62%
Proposed Study	X‐ray: 9544 CT‐scan: 8055	Image resizing to 100×100 pixels; image labeling; normalization; CNN based feature extraction; SMOTE‐ENN for data balancing; stratified 10‐fold CV.	CNN	X‐ray: 99.02%
				CT‐scan: 98.49%

The outcomes from prior efforts are summarized in Table 7. Notably [31], reported 100% accuracy, but this was based on only 1000 X‐ray and CT‐scan images. In contrast, the present study incorporates a substantially larger dataset, improving reliability. For ML and DL models, dataset size is critical, as greater data availability enhances algorithm performance.

Furthermore, research conducted in [22] also achieved 99% accuracy for X‐ray and CT‐scan data using the EfficientNet model. However, compared with such heavy architectures, the proposed 16‐layer CNN delivers similar accuracy while offering greater computational efficiency, ease of use, and interpretability. Additionally, studies summarized in Table 7, employed models such as InceptionV3, VGG, and SCovNet [30, 32, 33]. Therefore, this comparison demonstrates that the proposed methodology effectively supports COVID‐19 detection and severity assessment, enabling timely patient interventions.

For severity identification, the BIGDATA‐COVID19 prognostic dataset of 4995 samples from 1218 patients was used. To assess the proposed ERT model, results were compared with other studies employing the same dataset. Table 8 summarizes these works, including preprocessing methods, classifiers, and outcomes. As shown, the proposed framework outperforms prior approaches, achieving 98.00% accuracy with the ERT model. These findings confirm the effectiveness of the proposed framework method in accurately identifying severe COVID‐19 cases.

**Table 8:** Comparison of the prominent research works from the ‘BIGDATA‐COVID19 dataset’.

Study, Year	Methodology	Classifier	Result
Famiglini et al., 2021 [34]	Feature selection; SMOTE based data balancing; hyper‐parameter selection using the sequential model‐based optimization (SMBO) approach	Ensemble	AUC = 88.00%
Şiddeti et al., 2023 [35]	Filled missing values with kNN; min‐max normalization; SMOTE based data balancing	Adaboost	Accuracy = 89.54%
Proposed Study	Recursive Feature Elimination (RFE) for feature selection; standardization; SMOTENN‐based data balancing; stratified 10‐fold CV	Extra Randomized Tree Classifier	Accuracy = 98.00%

### 4.3 Limitations and Future Directions

The proposed smart lab framework shows strong potential but requires further validation before widespread hospital adoption. As the study relies on publicly available datasets, performance may vary with patients from different backgrounds and clinical settings. Practical deployment may also be affected by equipment availability, infrastructure support, and system compatibility. Moreover, strict data privacy and security measures will be essential when integrating AI and cloud‐based healthcare systems. Future work should include testing with real hospital data across larger patient groups to ensure reliability. Integration with IoT and cloud platforms could enhance real‐time monitoring and information sharing. Extending the framework to other infectious diseases and incorporating methods such as transfer learning or multimodal analysis would further increase its value and performance. Despite these challenges, the smart imaging lab addresses critical gaps in pandemic healthcare. By unifying RT‐PCR, X‐ray, CT‐scan, and blood tests, it reduces diagnostic delays, eases staff workload, and enables timely severity assessment. Its scalability and efficiency make it suitable even for resource‐limited hospitals, while its adaptability ensures long‐term relevance beyond COVID‐19.

## 5 Conclusion

The COVID‐19 pandemic has put immense pressure on the healthcare sector, underlining the need for practical and accurate diagnostic methods. In this study, a smart lab framework was introduced that combines RT‐PCR, X‐rays, CT‐scans, and blood tests within one platform to support early detection and severity assessment. The proposed approach not only streamlines the process but also provides dependable clinical support, reaching accuracy rates of 99.02% for X‐rays, 98.49% for CT‐scans, and 98.00% for severity classification. Moreover, the XAI technique Grad‐CAM was applied to highlight the affected lung regions, making the outputs easier to interpret by clinicians. Since the study integrates both imaging and blood data, it demonstrates how different diagnostic tools can be used together rather than in isolation. Thus, this work outlines a step toward diagnostic laboratories that are better prepared for future pandemics and new health challenges.

## Author Contributions

**Aditika Tungal:** conceptualization, data curation, formal analysis, methodology, writing – original draft, software. **Prabhsimran Singh:** investigation, methodology, writing – original draft, writing – review and editing. **Kuldeep Singh:** writing – review and editing, project administration, investigation, methodology. **Salil Bharany:** validation, investigation, writing – review and editing. **Ruby Pant:** visualization, validation, writing – review and editing. **Pankaj Deep Kaur:** conceptualization, writing – review and editing, resources. **Ajay Kumar:** writing – review and editing, investigation, data curation. **Ateeq Ur Rehman:** writing – review and editing, methodology, conceptualization. **Seada Hussen:** writing – review and editing, software, resources, methodology.

## Funding

The authors received no specific funding for this work.

## Ethics Statement

No animals or human subjects were involved in this study. The study utilized publicly available datasets, and all methods were carried out in accordance with relevant guidelines and regulations.

## Consent

The authors have nothing to report.

## Consent to Publish

The authors have nothing to report.

## Conflicts of Interest

The authors declare no conflict of interest.

## Clinical Trial Registration

As this study is not a clinical trial, so clinical trial registration details are not applicable.

## Transparency Statement

The lead authors, Ateeq Ur Rehman and Seada Hussen, affirm that this manuscript is an honest, accurate, and transparent account of the study being reported; that no important aspects of the study have been omitted; and that any discrepancies from the study as planned (and, if relevant, registered) have been explained.

## Supporting information


**Figure 1:** Accuracy Plot for X‐ray images. **Figure 2:** Performance metrics for X‐ray (a) Sensitivity (b) Specificity (c) Precision (d) FDR. **Figure 3:** Accuracy Plot for CT‐scan images. **Figure 4:** Performance metrics for CT‐scan (a) Sensitivity (b) Specificity (c) Precision (d) FDR. **Figure 5:** Accuracy of the various ML models in case of blood dataset. **Figure 6:** Performance metrics for Prognostic (a) Sensitivity (b) Specificity (c) Precision (d) FDR. **Table 1:** State‐of‐the‐art studies on COVID‐19 detection using chest radiography. **Table 2:** Prominent studies on COVID‐19 diagnosis using blood test values. **Table 3:** Image count in ‘Extensive COVID‐19 X‐ray and CT Chest Images Dataset’ from Mendeley. **Table 4:** List of blood parameters in the prognostic blood dataset.

## Data Availability

The dataset used in this study is publicly available at https://data.mendeley.com/datasets/8h65ywd2jr/3.
