# Supplementary material for: Artificial Intelligence Integrated Smart Medical Imaging Lab Framework for Enhanced Diagnosis and Treatment of Pandemic‐Prone Diseases
Source: Health Sci Rep. 2026 Mar 8;9(3):e71972. doi: 10.1002/hsr2.71972 (PMC12967674; doi:10.1002/hsr2.71972)
Supplement: Supplementary file 1 — Figure 1: Accuracy Plot for X‐ray images. Figure 2: Performance metrics for X‐ray (a) Sensitivity (b) Specificity (c) Precision (d) FDR. Figure 3: Accuracy Plot for CT‐scan images. Figure 4: Performance metrics for CT‐scan (a) Sensitivity (b) Specificity (c) Precision (d) FDR. Figure 5: Accuracy of the various ML models in case of blood dataset. Figure 6: Performance metrics for Prognostic (a) Sensitivity (b) Specificity (c) Precision (d) FDR. Table 1: State‐of‐the‐art studies on COVID‐19 detection using chest radiography. Table 2: Prominent studies on COVID‐19 diagnosis using blood test values. Table 3: Image count in ‘Extensive COVID‐19 X‐ray and CT Chest Images Dataset’ from Mendeley. Table 4: List of blood parameters in the prognostic blood dataset. [file HSR2-9-e71972-s001.docx]

**Supplementary Information**

| **Artificial Intelligence Integrated Smart Medical Imaging Lab Framework for Enhanced Diagnosis and Treatment of Pandemic-Prone Diseases** |
| --- |

S1. BACKGROUND

This section reviews approaches that support the development of smart laboratories. X-rays play an important role in detecting COVID-19 using machine-learning models. After identifying COVID-19 suspects via X-rays and CT-scans, patients undergo blood tests to determine infection severity. Hence, this section is divided into three sub-sections discussing the contributions of machine learning models in (a) establishing smart labs, (b) forecasting COVID-19 using X-rays and CT-scans, and (c) measuring infection severity through blood tests

***A. ESTABLISHMENT OF SMART LABS USING ARTIFICIAL INTELLIGENCE***

AI-based technologies have been a boon for the medical sector, particularly in mitigating the spread of infectious diseases. They have enhanced patient care and lightened the burden on medical personnel. Treatment has also become more efficient, with less manual work (1). The ongoing pandemic has placed considerable strain on doctors, lab technicians, and hospital staff to find advanced technological solutions (2). To handle the high influx of patients at testing laboratories and minimize virus contamination, the automation of smart labs is essential. This includes centralizing services such as X-rays, CT-scans, and blood tests in one location using machine learning and deep learning techniques (3). This approach enhances testing efficiency. Automated ML and DL can also handle complex data and devise intelligent solutions (4). These technologies streamline the process, starting with an initial test to determine COVID-19 status. Patients who test positive then undergo blood tests to check severity. In this way, the concept of automated smart labs provides a centralized setup that supports complete testing and better management of suspected cases.

B. PREDICTION OF COVID-19 USING X-RAYS AND CT-SCANS

Previous studies have demonstrated that chest X-ray is a fundamental imaging method for detecting COVID-19, owing to its cost-effectiveness, minimal radiation, rapid outcomes, and user-friendly nature (5,6). Along with RT-PCR, it can be used in the preliminary stage of infection to detect COVID-19 suspects. Researchers have made significant contributions in this domain, with some presented in Table 1.

Traditional X-rays and CT scans may have errors and require manual intervention; therefore, researchers have integrated deep learning (DL) techniques to swiftly identify traits from these images (7). Some studies focused only on chest X-ray datasets (8–10), reporting strong performance such as 98.00% accuracy (10). Others used CT images (11–14), with the highest accuracy of 99.12% (13). Moreover, (15) incorporated both X-ray and CT-scan datasets and achieved 89.50% accuracy using NasNet and Xception algorithms.

Table 1 summarizes the datasets, preprocessing steps, classifiers, and evaluation metrics used in these works. Overall, most studies achieved accuracy above 95.00% such as (11,12). However, many of these works were carried out on relatively small datasets, relied on only one type of image, or did not test their models across different populations. Notably, (15), who incorporated both X-ray and CT-scan datasets, reported a relatively lower accuracy of 89.50% compared to other studies. These gaps point to the need for approaches that use larger and more varied data and that can work reliably in broader clinical settings.

TABLE 1

State-of-the-art studies on COVID-19 detection using chest radiography

| Author, Year | Dataset | Image type | Output Class | Image Pre-Processing | Classifier | Results |
| --- | --- | --- | --- | --- | --- | --- |
| Rahimzadeh et al., 2021 (11) | **Created own data:**  COVID-CTset | CT-scan | COVID-19, Normal | Image resizing: [120,240] to [370,340]; Grad-CAM features | ResNet50V2 | Accuracy = 98.49% |
| Bhattacharyya et al., 2022 (8) | **Dataset 1:** Cohen | X-ray | COVID-19,  Pneumonia,  Normal | Image segmentation; data balancing; augmentation with affine transformations; histogram equalization; thresholding | VGG-19, BRISK, RF | Accuracy = 96.60%,  Sensitivity = 95.00%,  Specificity = 97.40% |
|  | **Dataset 2:** Pneumonia Dataset |  |  |  |  |  |
| Constantinou et al., 2023 (9) | Multiple datasets | X-ray | COVID-19, NON-COVID-19, NORMAL | ±10° rotation + flip; augmentation; 224×224 resize (ResNet, DenseNet) | ResNet101 | Accuracy = 96.00%,  Precision = 96.00%,  Recall = 96.00% |
| Gupta and Bajaj, 2023 (12) | SARS-CoV-2 chest CT-scan database | CT-scan | Covid, NonCovid | Image resizing: 256×256 (DarkNet19), 224×224 (MobileNetV2), 227×227 (new DL model) | DarkNet19 | Accuracy = 98.91%,  Sensitivity = 98.96%,  Specificity = 98.86%,  Precision = 98.88%,  F1-score = 99.00% |
| Albataineh et al., 2024 (13) | Multiple datasets | CT-scan | Normal, Mild, Moderate, Severe | CT slice filtering; multiple cropping | SVM | Accuracy = 99.12% |
|  |  |  |  |  |  | Accuracy = 98.24% |
|  |  |  |  |  |  | Accuracy = 98.73% |
|  |  |  |  |  |  | Accuracy = 99.90% |
| Amin et al., 2024 (10) | Chest X-ray data | X-ray | Pneumonia, COVID-19, Tuberculosis,  Normal | Resizing (100×100); grayscale; median/mean filtering; HOG + LBP feature extraction | [Ensemble Learning](https://www.sciencedirect.com/topics/computer-science/ensemble-learning) | Accuracy = 98.00% |
| Hassan et al., 2024 (14) | COVIDx CT dataset | CT-scan | Covid-19, Normal | Resize to 224×224×3; augmentation; DCNN feature extraction; normalization | ResNet50 | Accuracy = 99.07% |
| Ertam and Kilincer, 2025 (15) | X-Ray and CT-scan | X-ray and CT-scan | Normal, Infected | Random selection of 500 images per class | Xception, NasNet | Accuracy = 89.50% |

C. DETERMINING THE SEVERITY OF PATIENTS USING SEVERAL BLOOD TESTS

Routine blood tests serve as effective indicators for assessing the severity of COVID-19, offering a fast and affordable option compared to imaging methods (16). Clinical studies report abnormalities in several hematological and biochemical parameters of infected patients, making blood-based approaches suitable for early triage (17). Recent research using routine blood tests and machine learning shows varied but generally strong performance (see Table 2). (18) reported 88.00% accuracy using Random Forest, while (19) achieved 91.00% with logistic regression on emergency department cohorts. (20) further improved results to 92.00% with Random Forest and SMOTE-based balancing, and (21) obtained 85.90% sensitivity with Naïve Bayes. Additional studies demonstrated strong outcomes with diverse techniques: (22) used Random Forest to achieve 89.00% accuracy; (23) applied Gradient Boosting with up to 82.00% accuracy for mortality prediction; (24) explored survival analysis with Cox regression and survivalSVM, reaching an AUC of 77.80%; and (25) applied logistic regression with SMOTE balancing to obtain 82.00% accuracy.

Table 2 summarizes these contributions, highlighting datasets, preprocessing, feature selection, balancing strategies, and evaluation metrics. In general, most studies achieved performance between 85.00% and 92.00%, with some extending toward higher accuracies depending on dataset size and feature selection. However, results often relied on limited or single-hospital data, which restricts generalizability. This underlines the importance of developing approaches trained on larger and more diverse patient cohorts to ensure severity predictions that are reliable in broader clinical practice.

TABLE 2

Prominent studies on COVID-19 diagnosis using blood test values

| Author, Year | Dataset | No. of blood parameters | Pre-processing | Classifier | Result |
| --- | --- | --- | --- | --- | --- |
| Alves et al., 2021 (18) | Albert Einstein Hospital (São Paulo, Brazil) - Public Dataset | 23 | Missing values handled; irrelevant features removed; iterative imputer for balancing | Random Forest | Accuracy = 88.00%  Sensitivity = 66.00%,  Specificity = 91.00%,  F1-score = 76.00%,  AUC-ROC = 86.00% |
| Rahman et al., 2021 (19) | **Dataset 1:**  Emergency Department, Metropolitan Academic Hospital, Boston | 20 | Missing values handled with multiple regression; SMOTE for balancing | Logistic Regression | Accuracy = 91.00%,  Sensitivity = 91.00%,  Specificity = 78.00%,  F1-score = 92.00%,  Precision = 92.00% |
|  | **Dataset 2:**  Wuhan, China |  |  |  |  |
| Chadaga et al., 2022 (20) | Albert Einstein Hospital, Brazil (via Kaggle) | 14 | Zero-mean normalization; missing values reduced; new variable created; Pearson’s correlation coefficient (PCC) applied; RF & SHAP for feature importance; SMOTE balancing; grid search tuning | Random Forest | Accuracy = 92.00%,  F1-score = 83.00%,  AUC = 80.00,  Recall = 71.00% |
| Zhang et al., 2022 (21) | Shenzhen Third People's Hospital | 38 | Normalization; standardization; missing values handled with kNN interpolation | Naïve Bayes | Sensitivity = 85.90%,  Specificity = 75.00%,  AUC = 90.00% |
| N and S, 2023 (22) | San Raffaele Hospital | 15 | Missing data imputed with statistical mean; feature selection with RFE | Random Forest | Accuracy = 89.00%,  F1-score = 88.00%,  Precision = 89.00%,  Recall = 86.00% |
| Styrzynski et al., 2023 (23) | Maria Skłodowska-Curie Hospital, Zgierz, Poland | 20 | Missing values replaced by mean; feature selection with sequential feature selector | Gradient Boosting | Accuracy = 76.00% (positive and negative) |
|  |  |  |  |  | Accuracy = 82.00% (surviving versus deceased) |
| Qin et al., 2024 (24) | Xinhua Hospital, Shanghai Jiao Tong University School of Medicine | 30 | Handled missing values (>20% removed, <20% imputed); selected 34 features via Cox regression (30 blood parameters); dataset split into training, validation, test | Stepcox [both] and survivalSVM | C-index = 78.60%,  AUC = 77.80% |
| Xiaoyan et al., 2025 (25) | Beijing Shijitan Hospital | 8 | Stratification; statistical tests (chi-square, Mann–Whitney, ANOVA); feature selection (univariate, stepwise); SMOTE balancing; 10-fold CV | Logistic Regression | Accuracy = 82.00%,  AUC = 86.00%,  Sensitivity = 60.00%,  Specificity = 90.00% |

**S2. Dataset details**

1. **X-ray and CT-scan**

TABLE 3

Image count in ‘Extensive COVID-19 X-ray and CT Chest Images Dataset’ from Mendeley

| Total X-ray images | Total CT-scan images | | | COVID | | | Non-COVID | | |
| --- | --- | --- | --- | --- | --- | --- | --- | --- | --- |
| 9,544 | |  | | | | 4,044 | | 5,500 |  |
|  | | | 8,055 | | 5,427 | | | 2,628 | |

1. **Blood Data**

TABLE 4

List of blood parameters in the prognostic blood dataset

| Blood Parameter | Abbreviation | |
| --- | --- | --- |
| Mean Corpuscular Volume | | MCV |
| NeutrophilA | | NE |
| Platelets | | PLT |
| Red Blood Cells | | RBC |
| Mean Platelet Volume | | MPV |
| Mean Corpuscular Hemoglobin | | MCH |
| MonocyteB | | MOT |
| BasophilB | | BAT |
| Erythrocyte Distribution Width | | RDW |
| NeutrophilB | | NET |
| EosinophilA | | EO |
| Hemoglobin | | HGB |
| LymphocyteA | | LY |
| EosinophilB | | EOT |
| White Blood Cell | | WBC |
| BasophilA | | BA |
| Mean Corpuscular Hemoglobin Concentration | | MCHC |
| Hematocrit | | HCT |
| LymphocyteB | | LYT |
| MonocyteA | | MO |
| **Output parameter** | | **Severity** |

S3. MATHEMATICAL FORMULATION OF THE PROPOSED CNN

- **Input Layer:** The input is a 3D tensor $Ӽ\in\mathbb{R}^{100\times100\times3}$
- **First Convolutional Layer:** Ӽ1 = ReLU (Conv2D (Ӽ, W1) + b1)

where Conv2D (Ӽ, W1) = $\sum_{i=1}^{3} \sum_{j=1}^{3} \sum_{k=1}^{3} W_{1, i,j,k,m}. Ӽ_{h+i-1,w+j-1, k}$

such that W1 ∈ ℝ3×3×3×32

b1 ∈ R32

Ӽ1 ∈ ℝ100×100×32

- **Second Convolutional Layer:** Ӽ2 = ReLU (Conv2D (Ӽ1, W2) + b2)

where Conv2D (Ӽ1, W2) = $\sum_{i=1}^{3} \sum_{j=1}^{3} \sum_{k=1}^{32} W_{2, i,j,k,m}. Ӽ_{1, h+i-1,w+j-1, k}$

such that W2 ∈ ℝ3×3×32×32

b2 ∈ R32

Ӽ2 ∈ ℝ100×100×32

- **First MaxPooling Layer:** Ӽ3 = MaxPool2D (Ӽ2, (2,2))

where $Ӽ_{3,h,w,c}={max}_{i=1}^{2} {max}_{j=1}^{2} Ӽ_{2, 2h+i-1, 2w+j-1,c}$

such that Ӽ3 ∈ ℝ50×50×32

- **Third Convolutional Layer:** Ӽ4 = ReLU (Conv2D (Ӽ3, W3) + b3)

where Conv2D (Ӽ3, W3) = $\sum_{i=1}^{3} \sum_{j=1}^{3} \sum_{k=1}^{32} W_{3, i,j,k,m}. Ӽ_{3, h+i-1,w+j-1, k}$

such that W3 ∈ ℝ3×3×32×32

b3 ∈ ℝ32

Ӽ4 ∈ ℝ50×50×32

- **Fourth Convolutional Layer:** Ӽ5 = ReLU (Conv2D (Ӽ4, W4) + b4)

where Conv2D (Ӽ4, W4) = $\sum_{i=1}^{3} \sum_{j=1}^{3} \sum_{k=1}^{32} W_{4, i,j,k,m}. Ӽ_{4, h+i-1,w+j-1, k}$

such that W4 ∈ ℝ3×3×32×32

b4 ∈ R32

Ӽ5 ∈ ℝ50×50×32

- **Second MaxPooling Layer:** Ӽ6 = MaxPool2D (Ӽ5, (2,2))

where $Ӽ_{6,h,w,c}={max}_{i=1}^{2} {max}_{j=1}^{2} Ӽ_{5, 2h+i-1, 2w+j-1,c}$

such that Ӽ6 ∈ ℝ25×25×32

- **Fifth Convolutional Layer:** Ӽ7 = ReLU (Conv2D (Ӽ6, W5) + b5)

where Conv2D (Ӽ6, W5) = $\sum_{i=1}^{3} \sum_{j=1}^{3} \sum_{k=1}^{32} W_{5, i,j,k,m}. Ӽ_{6, h+i-1,w+j-1, k}$

such that W5 ∈ ℝ3×3×32×32

b5 ∈ R32

Ӽ7 ∈ ℝ25×25×32

- **Sixth Convolutional Layer:** Ӽ8 = ReLU (Conv2D (Ӽ7, W6) + b6)

where Conv2D (Ӽ7, W6) = $\sum_{i=1}^{3} \sum_{j=1}^{3} \sum_{k=1}^{32} W_{6, i,j,k,m}. Ӽ_{7, h+i-1,w+j-1, k}$

such that W6 ∈ ℝ3×3×32×32

b6 ∈ R32

Ӽ8 ∈ ℝ25×25×32

- **Third MaxPooling Layer:** Ӽ9 = MaxPool2D (Ӽ8, (2,2))

where $Ӽ_{9,h,w,c}={max}_{i=1}^{2} {max}_{j=1}^{2} Ӽ_{8, 2h+i-1, 2w+j-1,c}$

such that Ӽ9 ∈ ℝ12×12×32

- **Seventh Convolutional Layer:** Ӽ10 = ReLU (Conv2D (Ӽ9, W7) + b7)

where Conv2D (Ӽ9, W7) = $\sum_{i=1}^{3} \sum_{j=1}^{3} \sum_{k=1}^{32} W_{7, i,j,k,m}. Ӽ_{9, h+i-1,w+j-1, k}$

such that W7 ∈ ℝ3×3×32×32

b7 ∈ R32

Ӽ10 ∈ ℝ12×12×32

- **Eighth Convolutional Layer:** Ӽ11 = ReLU (Conv2D (Ӽ10, W8) + b8)

where Conv2D (Ӽ10, W8) = $\sum_{i=1}^{3} \sum_{j=1}^{3} \sum_{k=1}^{32} W_{8, i,j,k,m}. Ӽ_{10, h+i-1,w+j-1, k}$

such that W8 ∈ ℝ3×3×32×32

b8 ∈ R32

Ӽ11 ∈ ℝ12×12×32

- **Fourth MaxPooling Layer:** Ӽ12 = MaxPool2D (Ӽ11, (2,2))

where $Ӽ_{12,h,w,c}={max}_{i=1}^{2} {max}_{j=1}^{2} Ӽ_{11, 2h+i-1, 2w+j-1,c}$

such that Ӽ12 ∈ ℝ6×6×32

- **Dropout Layer:** Ӽ13 = Dropout (Ӽ12, 0.25)

such that Ӽ13 ∈ ℝ6×6×32 (during training, 25% of the values in Ӽ12 are set to 0)

- **Flatten Layer:** Ӽ14 = Flatten (Ӽ13)

such that Ӽ14 ∈ ℝ1152

- **First Dense Layer:** Ӽ15 = ReLU (W9 . Ӽ14 + b9)

where $W_{9 .}$ $Ӽ_{14}= \sum_{i=1}^{1152} W_{9,j,i}$. $Ӽ_{14,i}$

such that W9 ∈ ℝ64×1152

b9 ∈ ℝ64

Ӽ15 ∈ R64

- **Output Layer (Dense Layer with Softmax Activation):**

Ŷ = Softmax (W10 . Ӽ15 + b10)

where W10 . Ӽ15 = $\sum_{i=1}^{64} W_{10,j,i .}Ӽ_{15,i}$

and Softmax(z)i = $\frac{e^{z_{i}}}{\sum_{k=1}^{2} e^{z_{k}}}$

W10 ∈ ℝ2×64

b10 ∈ ℝ2

Ŷ ∈ ℝ2

The designed CNN processes input images of size 100×100×3, representing height, width, and color channels. Each convolutional layer applies filters (W) with biases (b) to the input (X) and uses ReLU activation (R). In the first layer, 32 filters of size 3×3 are used, producing 32 feature maps of 100×100. MaxPooling layers then reduce these maps to 50×50, preserving key features. Dropout layers randomly switch off 25% of neurons during training to limit overfitting. The flatten layer converts the three-dimensional feature maps into a one-dimensional vector of size 1152, which is processed by a dense layer with 64 neurons. The final dense layer employs a softmax activation function to generate a probability distribution over the two classes, COVID and Non-COVID, providing the model’s prediction. The network is trained using the binary cross-entropy loss function and the Adam optimizer, which adjusts weights (W) and biases (b) to minimize prediction error. A pseudo-algorithm (see section S4) is also included to outline the overall framework and the steps involved in completing the tasks.

**S4. PSEUDO ALGORITHM OF THE PROPOSED APPROACH**

**Pseudo Algorithm. COVID-19 Detection and Severity Assessment in Smart Lab Framework**

| Start  {  ***TASK 1: Determination of COVID-19***  ***Pre-requisites:*** RT-PCR report  if serious symptoms = = True:  then critical examination required;  **{**  ***Input***: X-ray and CT-scan image data  ***Processing:***  **{**  **image pre-processing ()**  image resizing to 100×100;  image labelling;  normalization;  **feature extraction ()**  CNN-based;  **data balancing ()**  SMOTE-ENN;  **data splitting ()**  test-train split;  **model evaluation ()**  stratified 10-fold cross validation;  **classification ()**  CNN-based;  **}**  ***Output:* COVID or Non-covid**  **}**  ***TASK 1*** fulfilled***;***  goto ***TASK 2****;*  ***TASK 2: Determination of severity among patients***  **{**  ***Input:*** Blood dataset (.csv)  ***Processing:***  **{**  **data pre-processing ()**  dropped null values;  standardization;  **feature selection ()**  recursive feature elimination (RFE);  **data balancing ()**  SMOTE-ENN;  **data splitting ()**  Test-train split;  **model evaluation ()**  stratified 10-fold cross validation;  **classification ()**  machine learning based;  **}**  ***Output:* Non-Severe or Severe**  **}**  ***TASK 2*** fulfilled;  **}**  stop |
| --- |

**S5. RESULTS AND DISCUSSIONS (X-ray)**


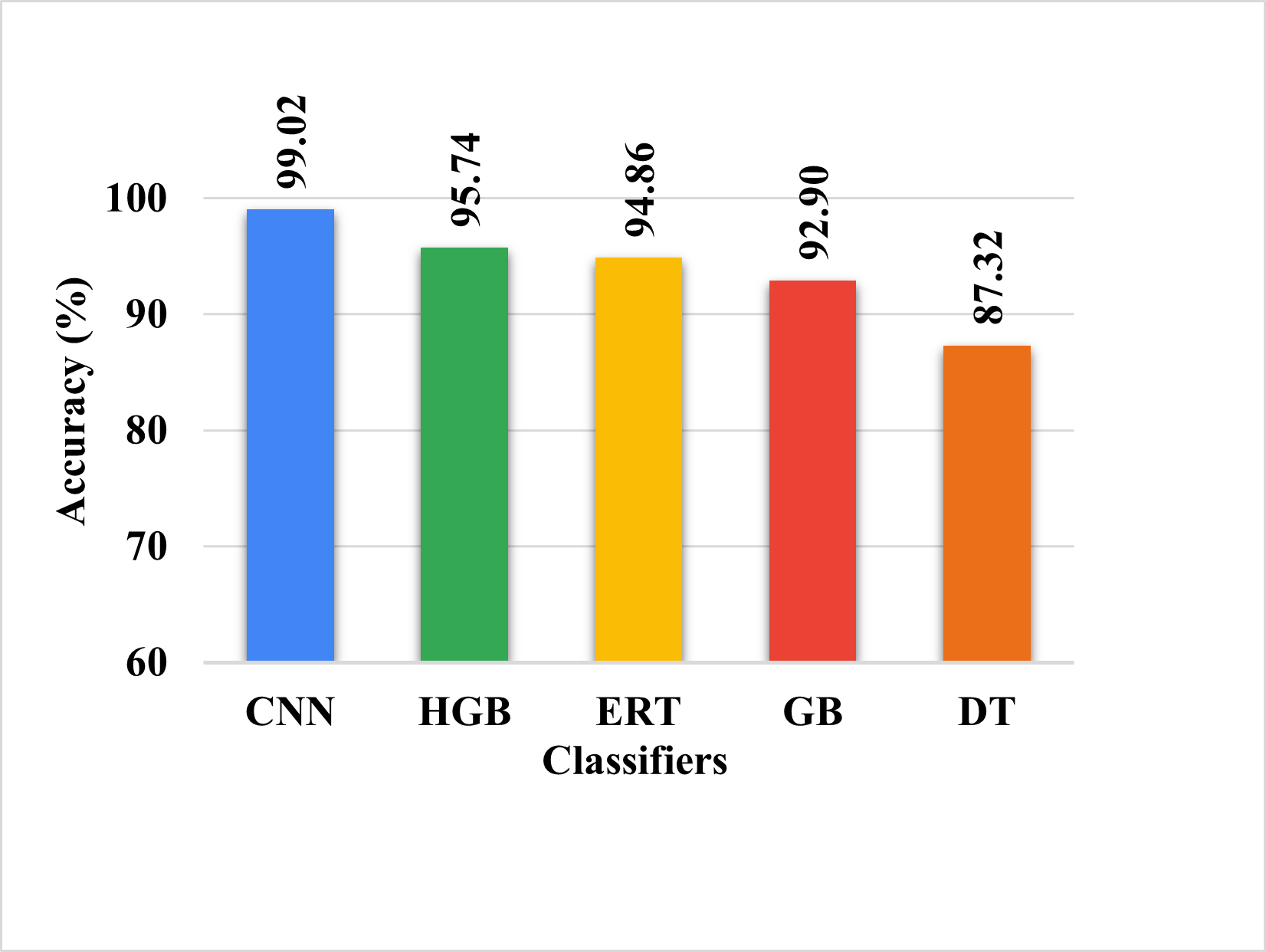


**FIGURE 1. Accuracy Plot for X-ray images**

In Figure 2, (a) represents sensitivity, (b) specificity, (c) precision, and (d) FDR. The first category corresponds to COVID, highlighted in light blue, while the second category reflects Non-COVID, colored in dark blue. For sensitivity, the CNN model demonstrates 99.00% accuracy for COVID and 99.04% for Non-COVID. In terms of specificity, the CNN model achieved 99.04% for COVID and 99.00% for Non-COVID. Precision is also highest for CNN, with 99.20% for COVID and 98.80% for Non-COVID. The FDR for the CNN model is 0.80% for COVID and 1.20% for Non-COVID, where lower values indicate better performance. Considering all these metrics, the CNN model exhibits superior performance among all classifiers. Among the other models, HGB demonstrates the second-best results, followed by ERT, GB, and DT, as illustrated in Figure 1.

| 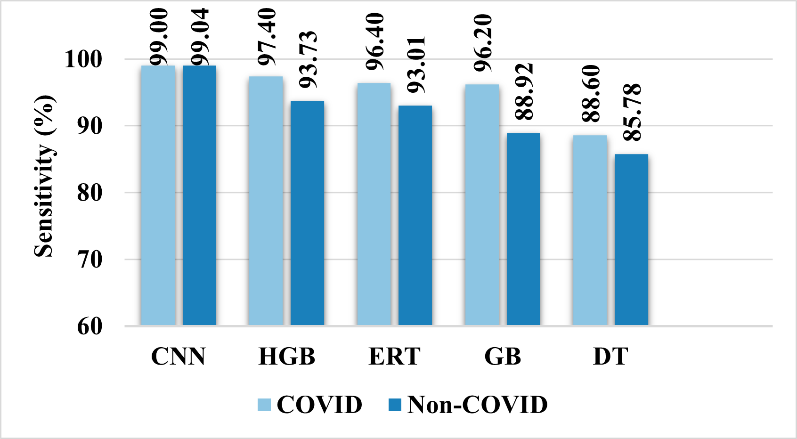   1. **Sensitivity** | 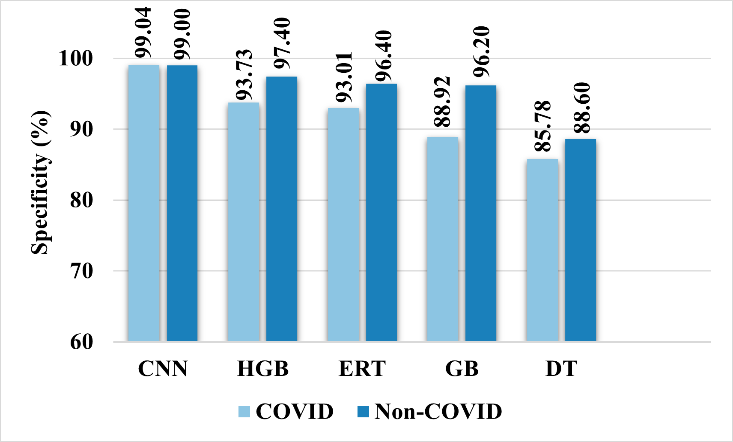   1. **Specificity** |
| --- | --- |
| 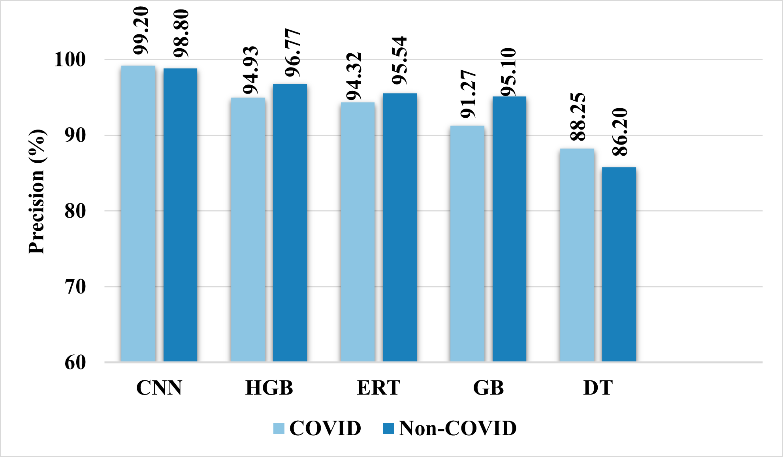   1. **Precision** | 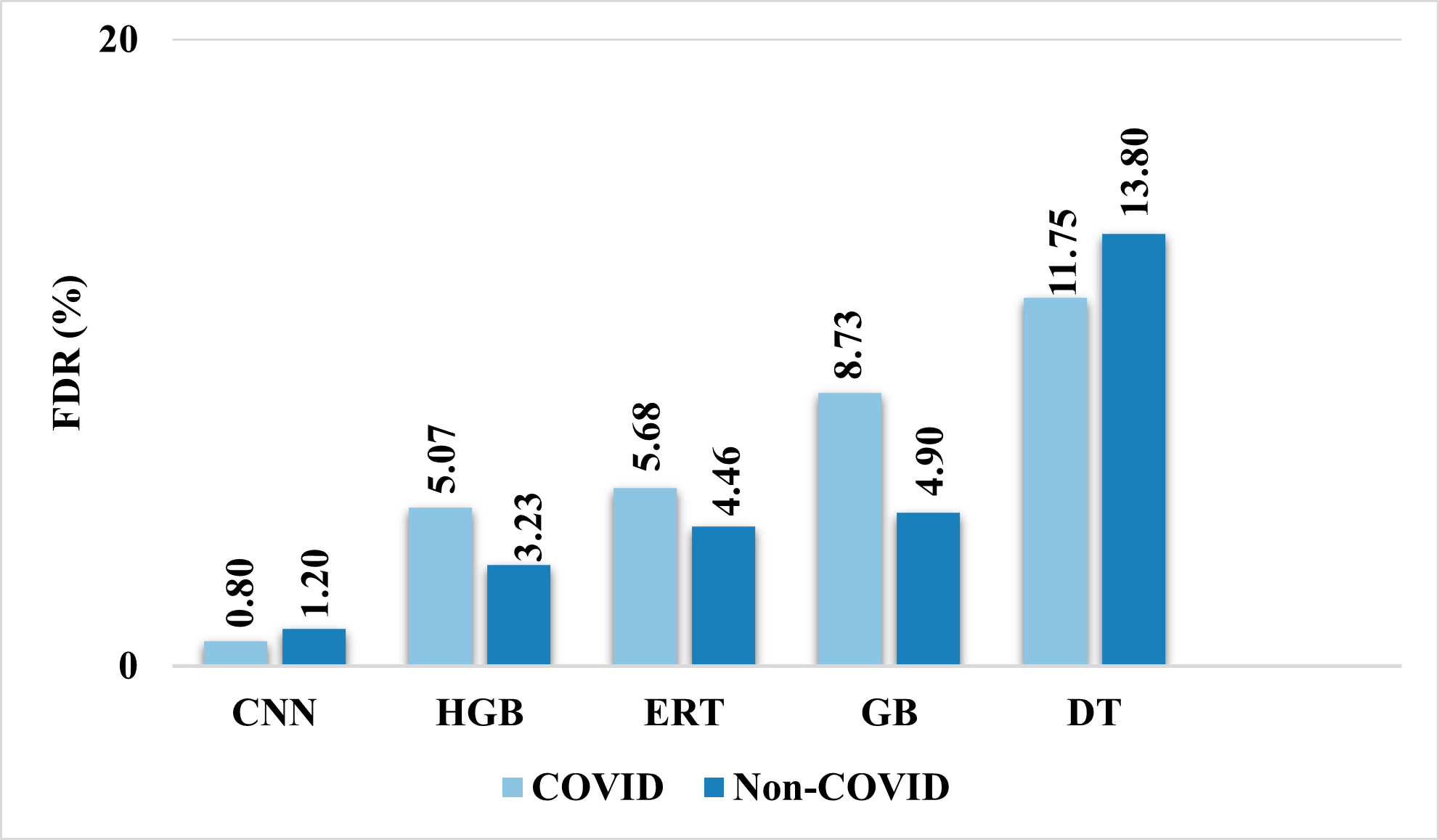   1. **FDR** |

**FIGURE 2. Performance metrics for X-ray (a) Sensitivity (b) Specificity (c) Precision (d) FDR**

**S6. RESULTS AND DISCUSSIONS (CT-scan)**


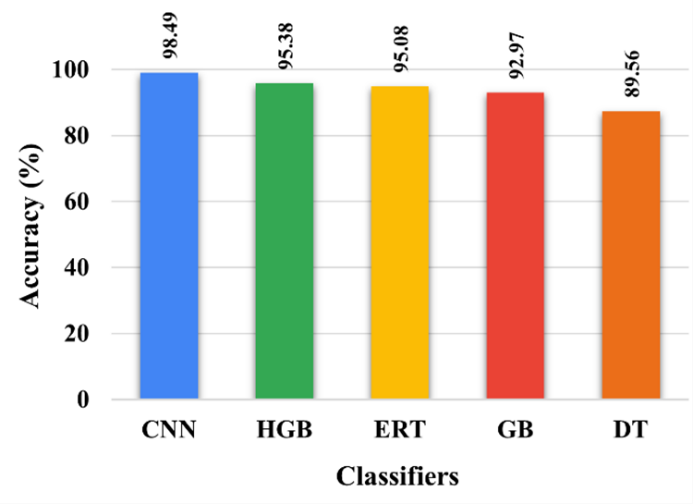


**FIGURE 3. Accuracy Plot for CT-scan images**

The model classifies images into COVID and Non-COVID categories. Class-wise performance is presented in Figure 4, where panel (a) represents sensitivity (%). The CNN model achieved the highest sensitivity, with 98.33% for COVID and 98.65% for Non-COVID.

| 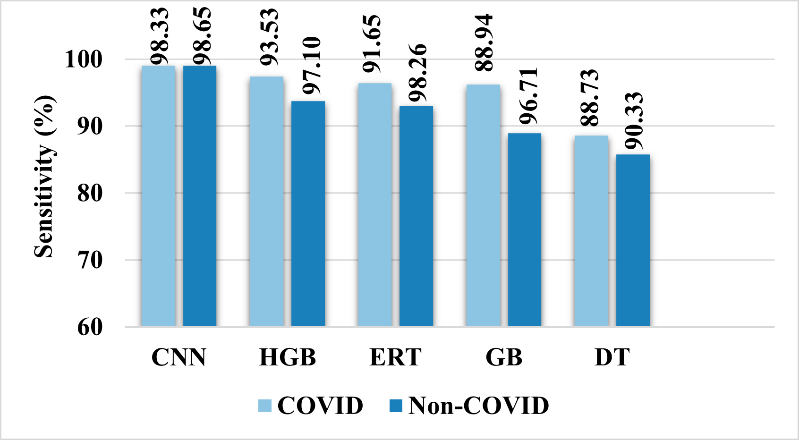   1. **Sensitivity** | 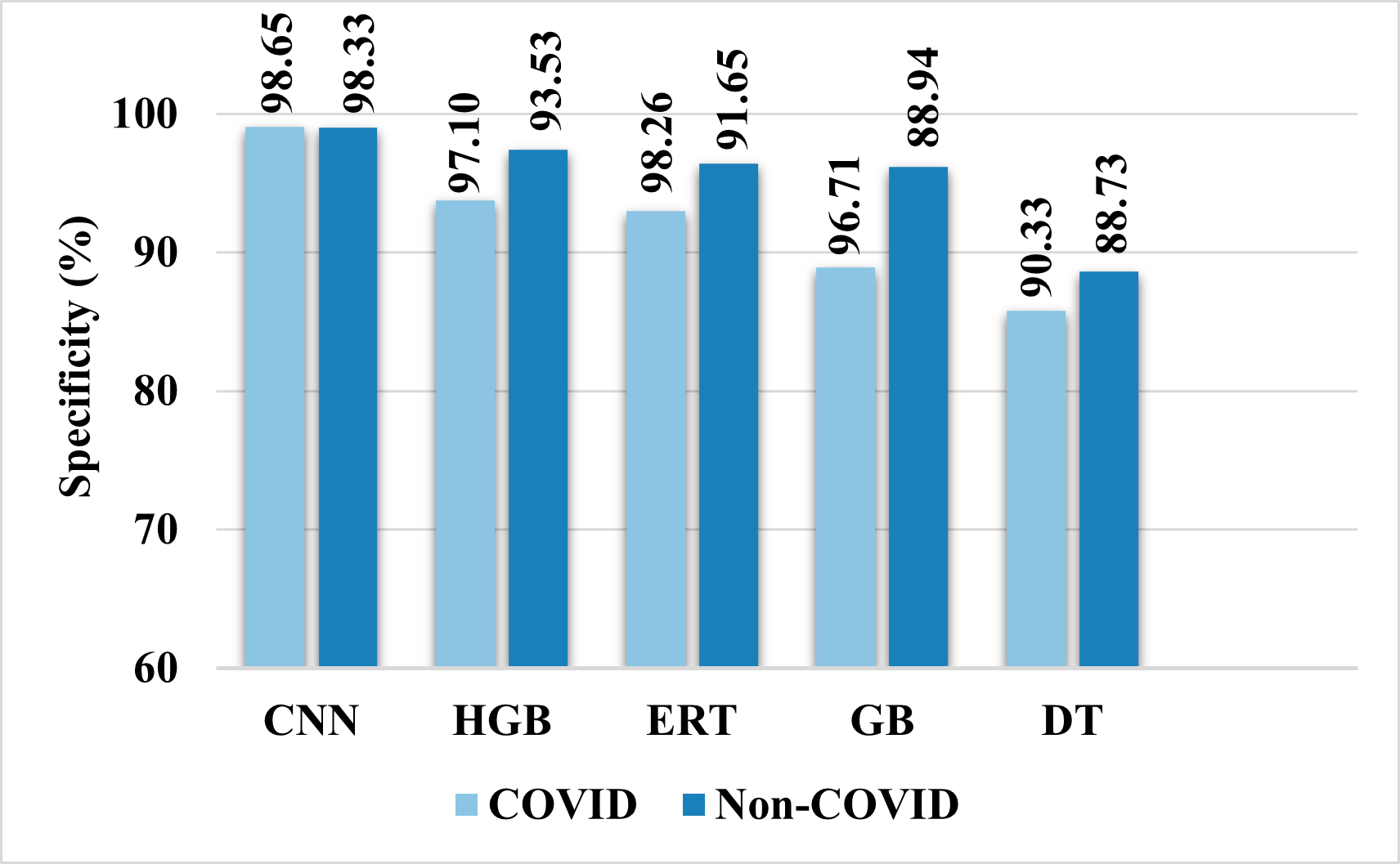   1. **Specificity** |
| --- | --- |
| 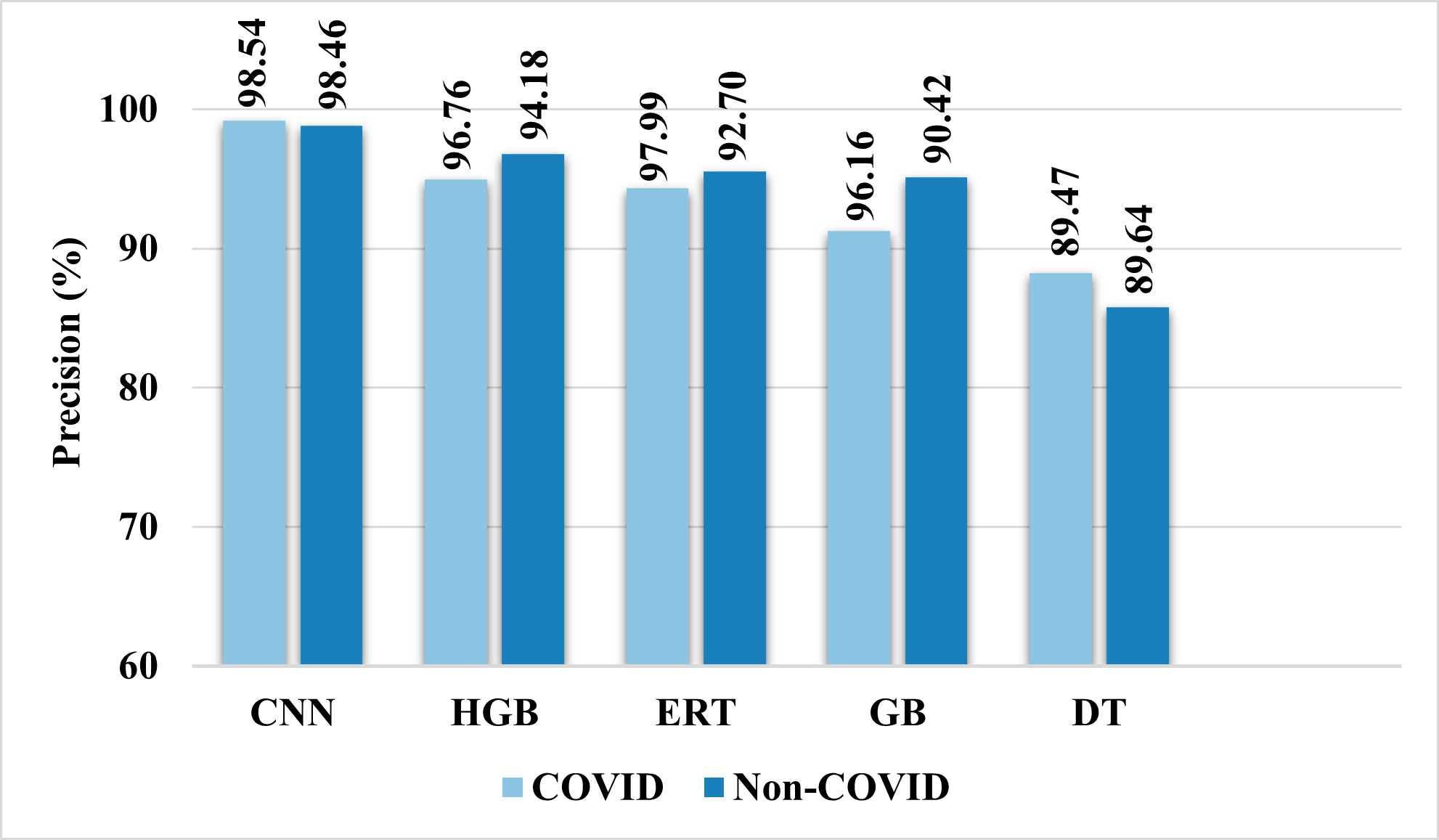   1. **Precision** | 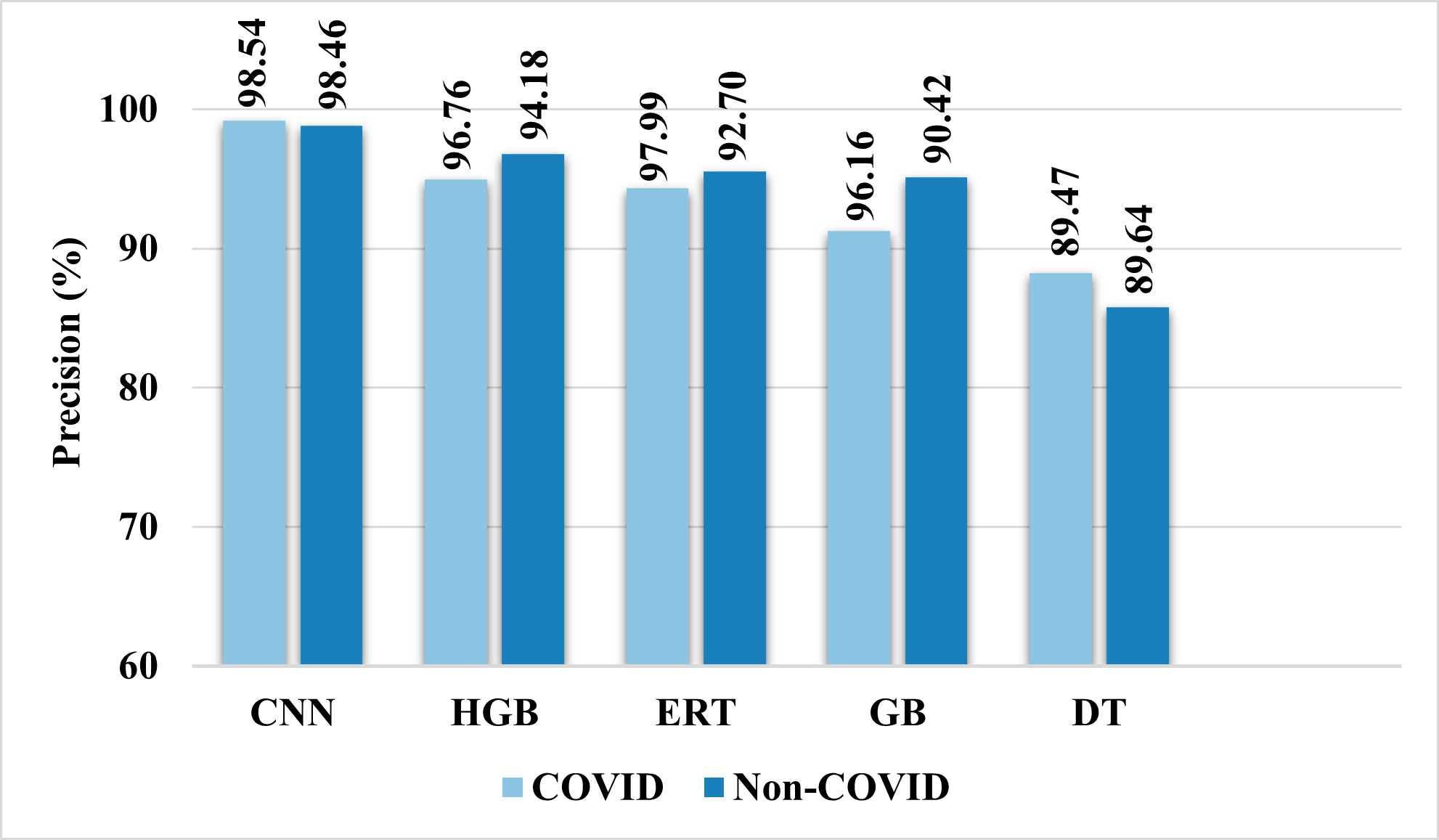   1. **FDR** |

**FIGURE 4. Performance metrics for CT-scan (a) Sensitivity (b) Specificity (c) Precision (d) FDR**

**S7. RESULTS AND DISCUSSIONS (Blood Dataset)**

**
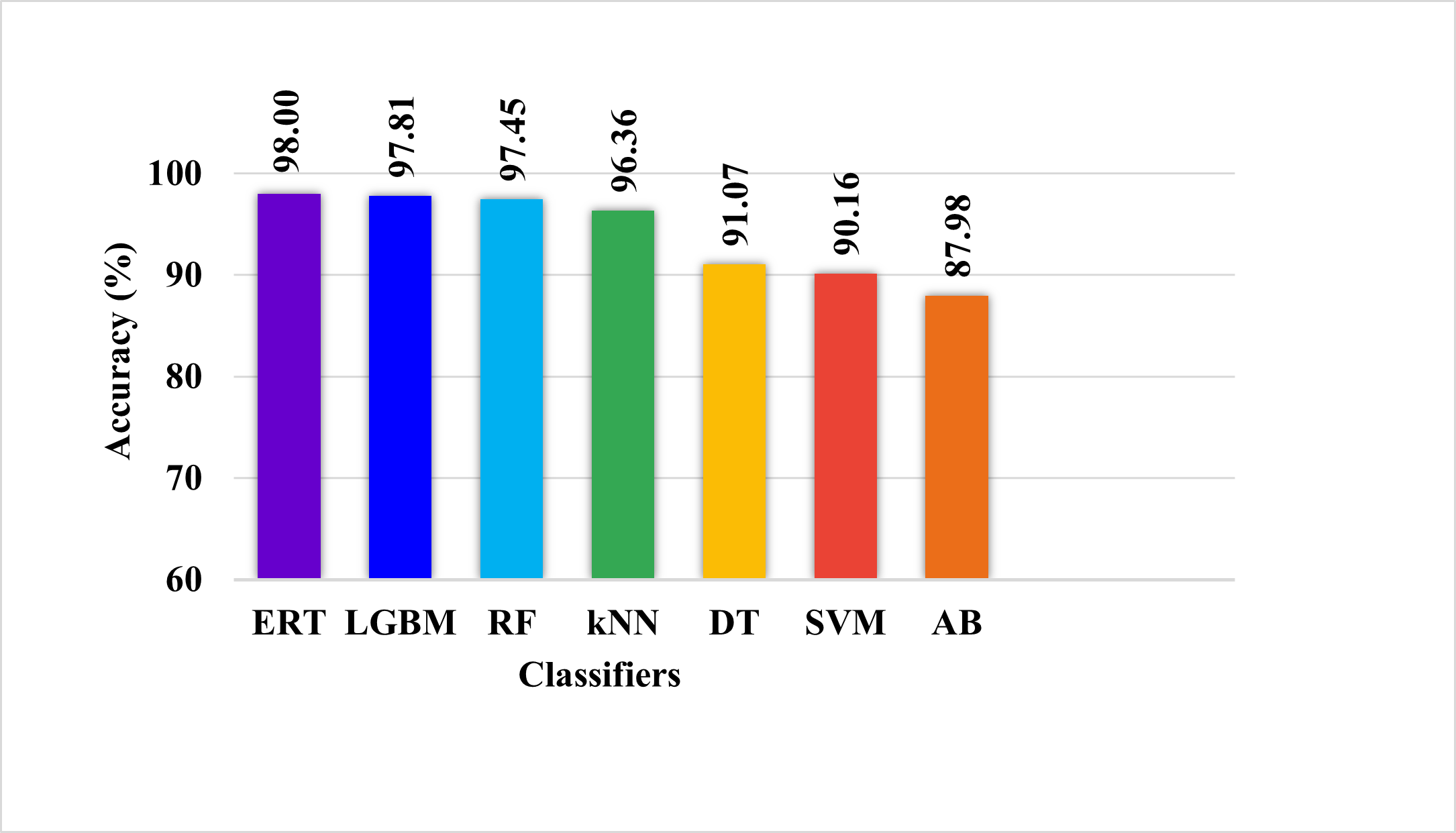
**

**FIGURE 5. Accuracy of the various ML models in case of blood dataset**

Figure 5 presents other performance metrics, including sensitivity, specificity, precision, and FDR (false discovery rate). The severe and non-severe classes are represented by dark blue and light blue, respectively. Furthermore Figure 6 displays the percentage values for each class, demonstrating that the ERT model outperforms the other classifiers across all metrics.

| 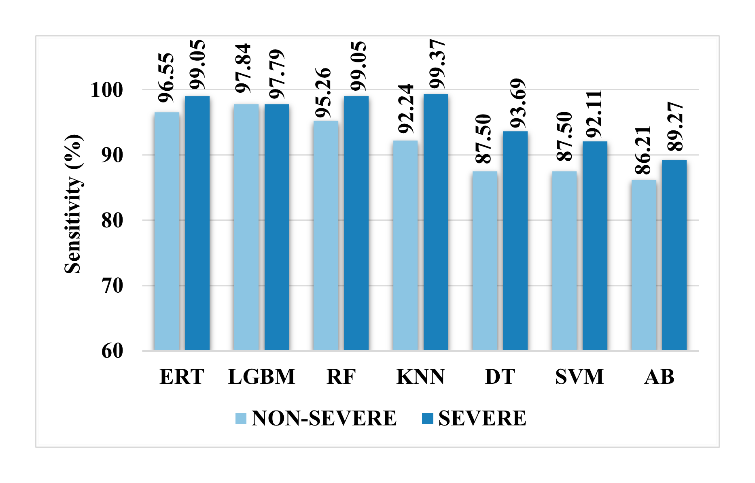   1. **Sensitivity** | 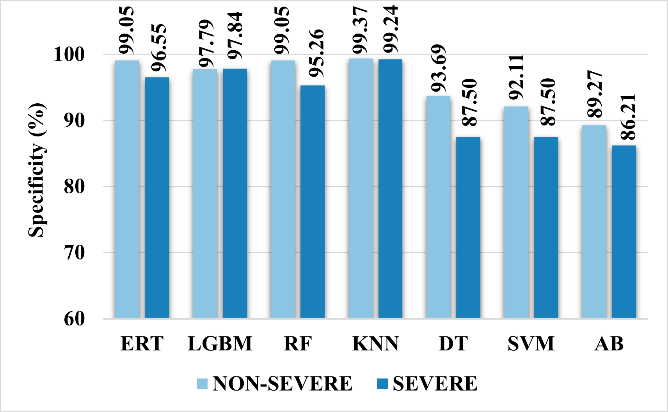   1. **Specificity** |
| --- | --- |
| 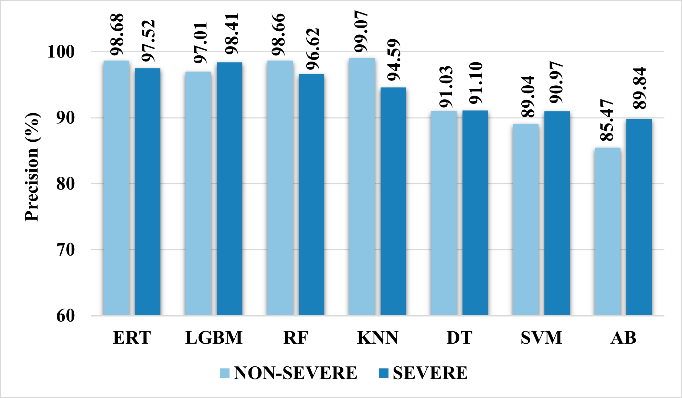   1. **Precision** | 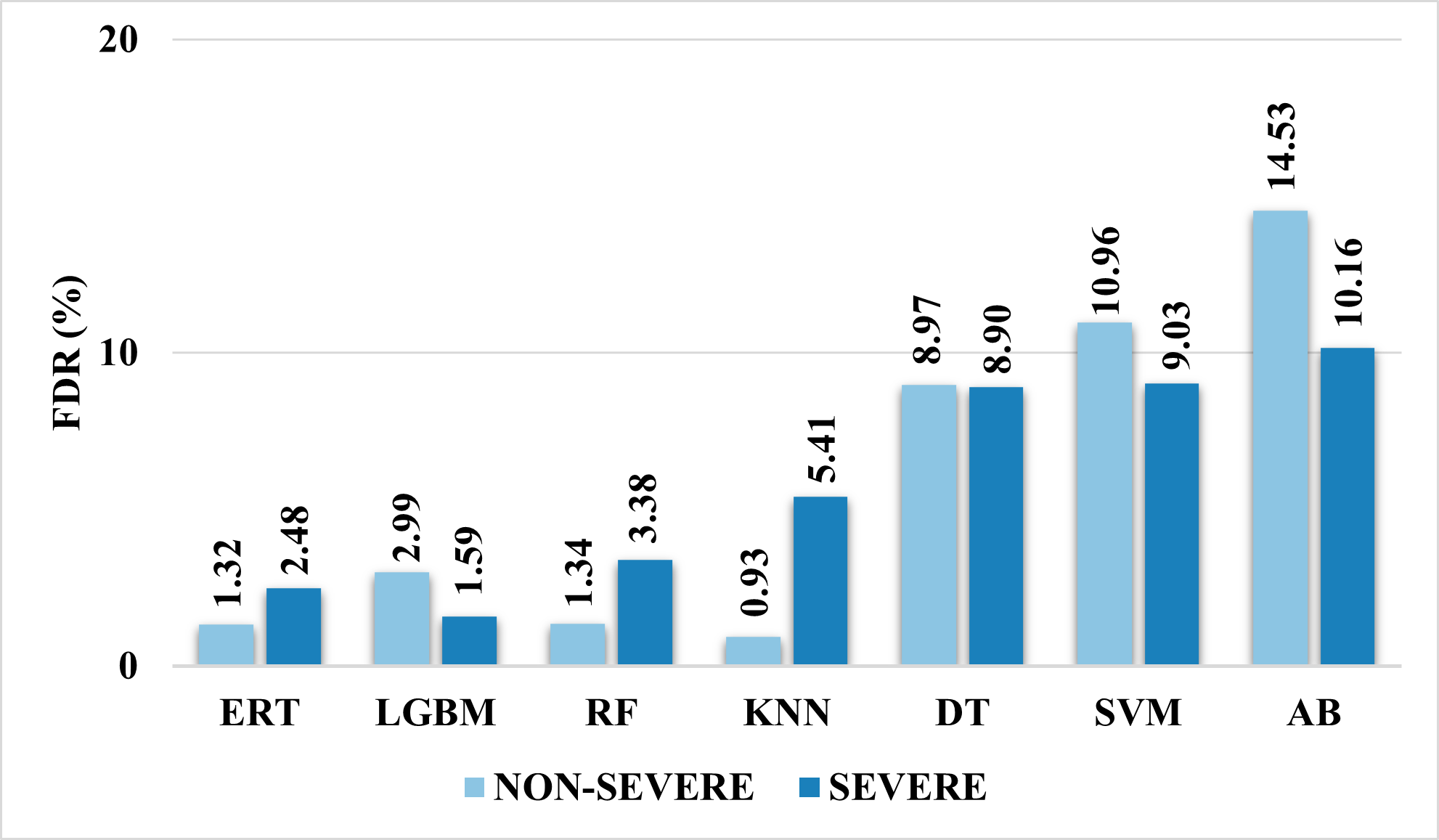   1. **FDR** |

**FIGURE 6. Performance metrics for Prognostic (a) Sensitivity (b) Specificity (c) Precision (d) FDR**

**References**

1. Ikemura K, Bellin E, Yagi Y, Billett H, Saada M, Simone K, et al. Using Automated Machine Learning to Predict the Mortality of Patients With COVID-19: Prediction Model Development Study. J Med Internet Res [Internet]. 2021 Feb 26;23(2):e23458. Available from: http://www.jmir.org/2021/2/e23458/

2. Mouliou DS, Pantazopoulos I, Gourgoulianis KI. COVID-19 Smart Diagnosis in the Emergency Department: all-in in Practice. Expert Rev Respir Med [Internet]. 2022 Mar 4;16(3):263–72. Available from: https://www.tandfonline.com/doi/full/10.1080/17476348.2022.2049760

3. Yang DM, Chang TJ, Hung KF, Wang ML, Cheng YF, Chiang SH, et al. Smart healthcare: A prospective future medical approach for COVID-19. J Chinese Med Assoc [Internet]. 2023 Feb 13;86(2):138–46. Available from: https://journals.lww.com/10.1097/JCMA.0000000000000824

4. Papoutsoglou G, Karaglani M, Lagani V, Thomson N, Røe OD, Tsamardinos I, et al. Automated machine learning optimizes and accelerates predictive modeling from COVID-19 high throughput datasets. Sci Rep [Internet]. 2021 Jul 23;11(1):15107. Available from: https://www.nature.com/articles/s41598-021-94501-0

5. Sahoo P, Saha S, Sharma SK, Mondal S, Gowda S. A Multi-stage framework for COVID-19 detection and severity assessment from chest radiography images using advanced fuzzy ensemble technique. Expert Syst Appl [Internet]. 2024 Mar;238:121724. Available from: https://linkinghub.elsevier.com/retrieve/pii/S0957417423022261

6. Miah J, Khan RH, Ahmed S, Mahmud MI. A comparative study of Detecting Covid 19 by Using Chest X-ray Images– A Deep Learning Approach. In: 2023 IEEE World AI IoT Congress (AIIoT) [Internet]. IEEE; 2023. p. 0311–6. Available from: https://ieeexplore.ieee.org/document/10174382/

7. Mangal A, Kalia S, Rajgopal H, Rangarajan K, Namboodiri V, Banerjee S, et al. CovidAID: COVID-19 Detection Using Chest X-Ray. 2020;1–10. Available from: http://arxiv.org/abs/2004.09803

8. Bhattacharyya A, Bhaik D, Kumar S, Thakur P, Sharma R, Pachori RB. A deep learning based approach for automatic detection of COVID-19 cases using chest X-ray images. Biomed Signal Process Control [Internet]. 2022 Jan;71:103182. Available from: https://linkinghub.elsevier.com/retrieve/pii/S1746809421007795

9. Constantinou M, Exarchos T, Vrahatis AG, Vlamos P. COVID-19 Classification on Chest X-ray Images Using Deep Learning Methods. Int J Environ Res Public Health [Internet]. 2023 Jan 22;20(3):2035. Available from: https://www.mdpi.com/1660-4601/20/3/2035

10. Amin SU, Taj S, Hussain A, Seo S. An automated chest X-ray analysis for COVID-19, tuberculosis, and pneumonia employing ensemble learning approach. Biomed Signal Process Control [Internet]. 2024 Jan;87:105408. Available from: https://linkinghub.elsevier.com/retrieve/pii/S1746809423008418

11. Rahimzadeh M, Attar A, Sakhaei SM. A fully automated deep learning-based network for detecting COVID-19 from a new and large lung CT scan dataset. Biomed Signal Process Control [Internet]. 2021 Jul;68:102588. Available from: https://linkinghub.elsevier.com/retrieve/pii/S1746809421001853

12. Gupta K, Bajaj V. Deep learning models-based CT-scan image classification for automated screening of COVID-19. Biomed Signal Process Control [Internet]. 2023;80(P1):104268. Available from: https://doi.org/10.1016/j.bspc.2022.104268

13. Albataineh Z, Aldrweesh F, Alzubaidi MA. COVID-19 CT-images diagnosis and severity assessment using machine learning algorithm. Cluster Comput [Internet]. 2024 Feb 24;27(1):547–62. Available from: https://link.springer.com/10.1007/s10586-023-03972-5

14. Hassan E, Shams MY, Hikal NA, Elmougy S. Detecting COVID-19 in chest CT images based on several pre-trained models. Multimed Tools Appl [Internet]. 2024 Jan 15; Available from: https://link.springer.com/10.1007/s11042-023-17990-3

15. Ertam F, Kilincer IF. Predicting and diagnosis of COVID-19 based on IoT and machine learning algorithm. In 2025. p. 255–90. Available from: https://linkinghub.elsevier.com/retrieve/pii/S0065245824000731

16. Fan Y, Liu M, Sun G. An interpretable machine learning framework for diagnosis and prognosis of COVID-19. Akbar S, editor. PLoS One [Internet]. 2023 Sep 21;18(9):e0291961. Available from: https://dx.plos.org/10.1371/journal.pone.0291961

17. Abbasi Habashi S, Koyuncu M, Alizadehsani R. A Survey of COVID-19 Diagnosis Using Routine Blood Tests with the Aid of Artificial Intelligence Techniques. Diagnostics. 2023;13(10).

18. Alves MA, Castro GZ, Oliveira BAS, Ferreira LA, Ramírez JA, Silva R, et al. Explaining machine learning based diagnosis of COVID-19 from routine blood tests with decision trees and criteria graphs. Comput Biol Med [Internet]. 2021 May;132:104335. Available from: https://linkinghub.elsevier.com/retrieve/pii/S0010482521001293

19. Rahman T, Al-Ishaq FA, Al-Mohannadi FS, Mubarak RS, Al-Hitmi MH, Islam KR, et al. Mortality Prediction Utilizing Blood Biomarkers to Predict the Severity of COVID-19 Using Machine Learning Technique. Diagnostics [Internet]. 2021 Aug 31;11(9):1582. Available from: https://www.mdpi.com/2075-4418/11/9/1582

20. Chadaga K, Prabhu S, Vivekananda Bhat K, Umakanth S, Sampathila N. Medical diagnosis of COVID-19 using blood tests and machine learning. J Phys Conf Ser [Internet]. 2022 Jan 1;2161(1):012017. Available from: https://iopscience.iop.org/article/10.1088/1742-6596/2161/1/012017

21. Zhang R, Xiao Q, Zhu S, Lin H, Tang M. Using different machine learning models to classify patients into mild and severe cases of COVID‐19 based on multivariate blood testing. J Med Virol [Internet]. 2022 Jan 25;94(1):357–65. Available from: https://onlinelibrary.wiley.com/doi/10.1002/jmv.27352

22. N HP, S R. Prediction of COVID-19 using Machine Learning Models based on Clinical Blood Test Data. Int Res J Adv Sci Hub [Internet]. 2023 May 28;5(Issue 05S):338–44. Available from: https://rspsciencehub.com/article_23845.html

23. Styrzynski F, Zhakparov D, Schmid M, Roqueiro D, Lukasik Z, Solek J, et al. Machine Learning Successfully Detects Patients with COVID-19 Prior to PCR Results and Predicts Their Survival Based on Standard Laboratory Parameters in an Observational Study. Infect Dis Ther [Internet]. 2023 Jan 4;12(1):111–29. Available from: https://link.springer.com/10.1007/s40121-022-00707-8

24. Qin Q, Li Q, Zhu G, Yu H, Peng M, Wu S, et al. Development of a COVID-19 early risk assessment system based on multiple machine learning algorithms and routine blood tests: a real-world study. Front Immunol [Internet]. 2024 Sep 30;15. Available from: https://www.frontiersin.org/articles/10.3389/fimmu.2024.1430899/full

25. Xiaoyan L, Zhongying B, Shuhong D, Jing S, Yijie Z, Jie Z, et al. Predictive model for coronavirus disease 2019 severity based on blood biomarkers: a retrospective study. Front Med [Internet]. 2025 Aug 8;12. Available from: https://www.frontiersin.org/articles/10.3389/fmed.2025.1597082/full
